# FAP^+^ pericyte-like cells promote monocyte differentiation into tumor-associated macrophages in glioblastoma

**DOI:** 10.1038/s12276-026-01827-8

**Published:** 2026-09-11

**Authors:** Magdalena Houdova Megova, Chloe Shard, Barbora Vymolova, Nikola Ternerova, Tereza Svablova, Tomas Buna, Daniel Straka, Zdislava Vanickova, Evzen Krepela, Helena Kupcova Skalnikova, Michal Kolar, Jana Sachova, Jan Kubovciak, Eva Balaziova, Petr Vymola, Petr Hrabal, Lisa M. Ebert, Ashwini Patil, Robert Tomas, David Netuka, Guillermo A. Gomez, Petr Busek, Aleksi Sedo

**Affiliations:** 1 Laboratory of Cancer Cell Biology, Institute of Biochemistry and Experimental Oncology, First Faculty of Medicine, Charles University, Prague, Czech Republic; 2 Centre for Cancer Biology, Adelaide University, Adelaide, South Australia Australia; 3 Laboratory of Genomics and Bioinformatics, Institute of Molecular Genetics of the Czech Academy of Sciences, Prague, Czech Republic; 4 Department of Pathology, Military University Hospital, Prague, Czech Republic; 5 Adelaide Medical School, University of Adelaide, Adelaide, South Australia Australia; 6 Cancer Clinical Trials Unit, Royal Adelaide Hospital, Adelaide, South Australia Australia; 7 Combinatics, Chiba, Japan; 8 Department of Neurosurgery, Motol and Homolka University Hospital, Prague, Czech Republic; 9 Department of Neurosurgery and Neurooncology, First Faculty of Medicine, Charles University and Military University Hospital, Prague, Czech Republic; 10 Present Address: Institute of Medical Biochemistry and Laboratory Diagnostics of the General University Hospital and the First Faculty of Medicine, Charles University, Prague, Czech Republic

**Keywords:** CNS cancer, Tumour immunology, Cancer microenvironment

## Abstract

Glioblastoma (GBM) is a highly aggressive primary brain tumor characterized by profound immunosuppression that facilitates tumor progression and promotes therapeutic resistance. Fibroblast activation protein (FAP), a recognized theranostic target in multiple cancers, is upregulated in GBM and predominantly expressed by pericyte-like stromal cells. Here we identify a role for FAP⁺ pericyte-like cells in shaping the GBM immune microenvironment through monocyte recruitment and differentiation. Analysis of The Cancer Genome Atlas (TCGA) datasets, supported by reverse-transcription quantitative PCR and immunohistochemistry, revealed that elevated FAP expression—serving as a proxy for the abundance of FAP⁺ pericyte-like cells—is associated with an immune-enriched tumor microenvironment characterized by higher macrophage abundance and elevated expression of M2 polarization markers. Spatial analyses, including immunofluorescence and spatial transcriptomics, demonstrated that immunosuppressive macrophages preferentially localize in proximity to FAP⁺ pericytes. Single-cell RNA sequencing identified these FAP⁺ cells as a distinct perivascular stromal subset with a unique expression pattern of extracellular matrix components and cytokines, including CCL2 and CSF1, with corresponding receptors expressed on myeloid cells. Functional assays using patient-derived FAP⁺ pericyte-like cells confirmed their ability to attract monocytes via soluble mediators and to promote their differentiation and polarization into tumor-associated macrophages with immunoregulatory features, partly mediated by the CSF1–CSF1R axis. Orthotopic co-implantation experiments in mice further supported their capacity to enhance myeloid infiltration in vivo. Consistent with these biological effects, a transcriptional signature characteristic of FAP⁺ pericytes correlated with worse overall survival in patients with GBM. Together, these findings position FAP⁺ pericyte-like cells as modulators of the GBM immune landscape, fostering a tumor-permissive niche by promoting the differentiation of circulating monocytes into immunoregulatory macrophages. Targeting this stromal population may offer new therapeutic avenues to reprogram tumor-associated immune responses in GBM.

## FAP pericytes shape immune landscape in GBM

Glioblastomas (GBMs), aggressive brain tumors, are marked by a complex microenvironment with diverse immune cells, including tumor-associated macrophages. This study explores the role of fibroblast activation protein (FAP)-positive pericyte-like cells in shaping the immune landscape of GBM. Using data from The Cancer Genome Atlas, single-cell RNA sequencing and spatial transcriptomics, the authors found that FAP^+^ pericytes are linked to increased monocyte recruitment and differentiation into tumor-associated macrophages, particularly those with an M2-like, tumor-supportive phenotype. These cells express high levels of chemokines such as CCL2 and CSF1, which are crucial for monocyte attraction and macrophage polarization. The study suggests that FAP^+^ pericytes contribute to the immunosuppressive environment in GBMs, potentially impacting patient survival. Future research could explore targeting these pericytes to modulate the tumor microenvironment and improve therapeutic outcomes. This summary was initially drafted using artificial intelligence, then revised and fact-checked by the author.

## Introduction

Glioblastomas (GBMs) are the most common and highly aggressive primary brain tumors in adults, characterized by a high degree of inter- and intratumoral heterogeneity, rapid and infiltrative growth, rampant but defective vasculature and profound local immunosuppression^1^. Central to these GBM features is the presence of diverse leukocyte subpopulations, notably a high proportion of tumor-associated macrophages (TAMs)^2^.

TAMs in GBM originate from microglia and, to a large extent, from blood-derived monocytes^2,3^ recruited by different soluble mediators such as MCP-1 (CCL2; monocyte chemotactic protein 1, chemokine (C–C motif) ligand 2), M-CSF (CSF1; macrophage colony-stimulating factor), GM-CSF (CSF2; granulocyte-macrophage colony-stimulating factor), HGF (hepatocyte growth factor), MIF (macrophage migration inhibitory factor) and SDF-1 (stromal cell-derived factor)^4,5^. During diapedesis through the endothelial layer and under the influence of factors in the perivascular niche, monocytes begin to differentiate and polarize into various TAM subpopulations^6^. The phenotype of these TAMs combines features of both classically activated proinflammatory M1 macrophages and alternatively activated anti-inflammatory M2 macrophages^7,8^.

Nonmalignant mesenchymal cells, such as cancer-associated fibroblasts (CAFs), constitute an essential component of the tumor microenvironment (TME) in extracranial malignancies and contribute to several hallmarks of cancer^9^. CAFs originate from local mesenchymal cells, such as fibroblasts and pericytes, or nonlocal sources, such as bone marrow-derived mesenchymal cells^9^. Despite their heterogeneity, CAFs frequently express fibroblast activation protein (FAP), a serine protease that is highly expressed across many tumor types but shows limited expression in most healthy adult tissues^10,11^. This tumor-associated expression pattern has made FAP an attractive theranostic target in both intra- and extracranial malignancies^12^. Among their tumor-promoting functions, FAP^+^ CAFs play an essential role in shaping the tumor immune landscape in extracranial tumors^13^. However, whether analogous FAP⁺ stromal populations exert similar immunomodulatory functions in GBM remains unclear, given the unique cellular composition and immune environment of the brain^14^.

Our previous studies have demonstrated FAP overexpression in GBM, particularly in the mesenchymal subtype^15,16^. Nonmalignant mesenchymal cells resembling pericytes and/or CAFs account for most FAP-expressing cells in GBM, although FAP can also be expressed by a subpopulation of tumor cells and by tumor endothelial cells^15–17^. Notably, the highest expression of FAP was observed in regions identified as hyperplastic blood vessels and microvascular proliferation in human GBMs^18^. We showed that FAP^+^ pericyte-like cells support GBM vascularization by enhancing endothelial cell migration and sprouting. In addition, they promote glioma cell migration and proliferation^19^.

Here, we aim to elucidate the relationship between FAP^+^ pericyte-like cells and immune myeloid cell subpopulations in the GBM microenvironment. Using in silico analysis of The Cancer Genome Atlas (TCGA) data, quantitative reverse-transcription PCR (RT–qPCR) and immunohistochemistry analysis of GBM tissues, we found that monocytes/macrophages are enriched in GBMs with high FAP expression. Furthermore, by using single-cell RNA sequencing (scRNA-seq), spatial transcriptomics and functional experiments, we found that FAP^+^ pericyte-like cells contribute to the migration and differentiation of monocytes into TAMs in perivascular regions. We propose that FAP^+^ pericytes play an important role in shaping the immunosuppressive properties in GBM.

## Materials and methods

### Patients

The study was approved by the Institutional Ethics Committee (study approval numbers 108-39/4-2014-UVN and 7/8/2014-25) and conducted in accordance with the Helsinki Declaration. Fully informed consent was obtained from all donors before neurosurgical resection. GBM diagnosis was established according to the 2021 WHO classification. Patients with newly diagnosed GBM (IDH-wild-type, grade 4 glioma, *n *= 117) undergoing neurosurgical resection at the Department of Neurosurgery, Na Homolce Hospital, Prague or at the Department of Neurosurgery and Neurooncology, First Faculty of Medicine, Charles University and Military University Hospital, Prague, were included in the study.

### In silico bioinformatic analysis

TCGA gene expression data from Affymetrix expression array platform HG-U133A were accessed via the GlioVis online portal (http://gliovis.bioinfo.cnio.es/) in September 2019^20^. For in silico analysis, data from newly diagnosed IDH-wild-type GBMs were divided into terciles based on FAP messenger RNA expression (FAP high = upper tercile, *n* = 119; FAP low = lower tercile, *n *= 119). To assess the cellular composition of the TME, we estimated the absolute abundance of immune and stromal cell populations in IDH-wild-type TCGA-GBM samples using the MCP-counter algorithm^21^, implemented via the R package immunedeconv. To evaluate the association between FAP expression and the TME landscape, patients were stratified into terciles based on their FAP expression levels. Abundance scores were calculated according to the MCP-counter framework based on log_2_-transformed gene expression values. In this study, the MCP-counter ‘cancer-associated fibroblast (CAF)’ category was used and referred to as nonmalignant mesenchymal cells. Ivy Glioblastoma Atlas (http://glioblastoma.alleninstitute.org/) was used to explore the expression of monocyte and macrophage markers in perivascular regions based on FAP mRNA expression. For this purpose, we defined ‘perivascular regions’ as Hyperplastic blood vessels and microvascular proliferations, divided them based on FAP expression into terciles, and compared the expression of monocyte and macrophage markers between ‘FAP-high’ (upper tercile) and ‘FAP-low’ (lower tercile) perivascular regions. To further identify the characteristics of FAP-high perivascular regions, differentially expressed genes (DEGs) between FAP-high and FAP-low regions were identified and subjected to gene set enrichment analysis using the iDEP application (http://bioinformatics.sdstate.edu/idep/).

### Isolation and quantification of total RNA, determination of RNA integrity and library preparation for RNA-bulk transcriptome analysis

Total RNA was isolated from 13 GBM tissues using RNeasy Micro Kit (Qiagen) according to the manufacturer’s protocol, including treatment by DNase I. The quantity and quality of isolated RNA were measured using a NanoDrop ND-1000 (Thermo Fisher Scientific) and Agilent 2100 Bioanalyzer (Agilent Technologies). The RNA integrity number ranged between 6.3 and 9.5. A KAPA mRNA HyperPrep kit with poly(A) mRNA selection (Roche) was used to construct the sequencing library, starting with 1 μg of total RNA.

### RNA-bulk transcriptome analysis

Libraries were sequenced on a NextSeq 500 platform (Illumina) using the 75-bp single-end configuration. The sequencing yielded an average of 30 million reads per sample. Technical quality control and gene quantification were done using the nf-core/RNAseq v1.4.2 bioinformatics pipeline^22^, with HISAT2 mapping^23^ and read counting with featureCounts^24^. GRCh38 (Ensembl annotation version 95) was chosen as the reference genome^25^. Gene set enrichment analysis was performed against Gene Ontology (GO) terms^26^ using the fgsea package^27^.

### Immunohistochemistry

CD45 and CD68 immunopositivity was detected in 4-µm formalin-fixed paraffin-embedded GBM tissue sections using primary monoclonal antibodies: anti-CD45 (clone X16/99, Leica), anti-CD68 (clone KP1, Thermo Fisher Scientific) following antigen retrieval using an EDTA-based pH 9.0 epitope retrieval solution (BOND Epitope Retrieval Solution 2) for 20 min. Bond polymer refine detection (Leica) was used to visualize the primary antibody signal and hematoxylin nuclear counterstaining. Images were captured by an experienced pathologist (Petr Hrabal) on an Axioskop 2 mot plus microscope using the Axiocam ICc1 camera (Zeiss). Using ImageJ^28^, the percentage of CD45 and CD68 positive area was analyzed in five random fields of view. FAP expression in stromal cells was evaluated by immunohistochemistry in a previous study^19^. The immunopositivity score for each tumor represents an average of five independent microscopic fields, evaluated using a four-tiered semiquantitative scale (where 0 is negative; 1 is perivascular positivity in sporadic blood vessels; 2 is perivascular positivity in over 2/3 of the visual field; 3 is extensive perivascular positivity and FAP^+^ trabeculae)^19^.

### Immunofluorescence

Immunofluorescence labeling was performed in 10-µm frozen sections of human GBM and mouse brain tumors. Samples were fixed and permeabilized using either a 1:1 mixture of methanol and acetone (5 minutes, −20 °C) or 4% paraformaldehyde followed by 0.1% Triton-X100. Nonspecific antibody binding was blocked with 10% fetal bovine serum (FBS) and 1% bovine serum albumin in Tris-buffered saline (60 min, room temperature). Human GBM sections were subjected to sequential double immunofluorescence labeling for FAP in combination with CD68, CD163, CD206, αSMA, PDGFRβ, NG2 or GFAP using the following primary antibodies diluted in Tris-buffered saline with 1% bovine serum albumin: anti-FAP (clone D8, Applied DNA Sciences, 1:800, 1 h, room temperature), anti-CD68 (clone D4B9C, Cell Signalling, 1:800, overnight, 4 °C), anti-CD163 antibody (clone GHI/61, Santa Cruz, 1:200, overnight, 4 °C) and anti-CD206 (clone 15-2, Santa Cruz, 1:200, overnight, 4 °C), anti-αSMA (clone 1A7, Abcam; 1:200; overnight, 4 °C), anti-PDGFRβ (clone PR72112, Invitrogen; 1:100; overnight, 4 °C), anti-NG2 (clone E3B3G, Cell Signaling; 1:200; overnight, 4 °C) and anti-GFAP (clone GF-01, Exbio; 1:200; overnight, 4 °C). Mouse tumor sections from the co-implantation model were stained for leukocyte and macrophage markers using anti-CD45 (clone 30-F11, R&D Systems; 1:100; overnight, 4 °C) and anti-F4/80 (clone CI-A3-1, Novus Biologicals; 1:500; overnight, 4 °C). Primary antibodies were visualized using the species-appropriate Alexa Fluor-conjugated secondary antibodies (anti-rat Alexa Fluor 488, anti-rabbit Alexa Fluor 594 or 488, anti-mouse Alexa Fluor 647 or 488, all 1:500, 60 min, room temperature). Images of human GBM sections were acquired using a Leica Stellaris 5 confocal microscope (Leica), whereas mouse tumor sections were imaged using an Olympus IX70 microscope equipped with an ORCA-Flash 4.0 camera (Hamamatsu). For mouse tumors, five representative fields per marker were imaged and positive cells were manually quantified using the Cell Counter plugin in ImageJ (NIH).

### Isolation and quantitation of total RNA and determination of RNA integrity for real-time qRT–PCR

Total RNA was isolated with the TRIzol reagent (Thermo Fisher Scientific) according to the manufacturer’s protocol. RNA concentration was assayed with the RiboGreen RNA quantitation Kit (Thermo Fisher Scientific) according to the manufacturer’s protocol. Integrity of the isolated RNA samples was determined using Agilent 2100 Bioanalyzer (Agilent Technologies) and the Agilent RNA 6000 Nano Kit (Agilent Technologies). All samples used for the real-time RT–PCR quantitation of the studied mRNAs displayed RNA integrity number ≥5.

### Real-time qRT–PCR

The forward and reverse primers and TaqMan probes (Thermo Fisher Scientific; Supplementary Table 1) were designed with the Primer Express software (Thermo Fisher Scientific). A two-step real-time RT–PCR assay was used to quantitate mRNA expression. SuperScript IV VILO MasterMix (Thermo Fisher Scientific) was used for reverse transcription according to the manufacturer’s instructions. The subsequent PCR step was performed with an aliquot of the RT-mix, corresponding to a PCR input of 50 ng of total RNA. The PCR assays for RT-samples were run in triplicate, along with no-template controls, on the QuantStudio 12K Flex instrument (Thermo Fisher Scientific). Threshold cycle (Ct) values for amplification reactions were determined from plots of background-subtracted fluorescence intensity (ΔFI) of the reporter dye (6-FAM) versus PCR cycle number using the QuantStudio 12K Flex Software. Expression data were normalized to a reference transcript using the ΔCt method (that is, 2^−ΔCt^).

### Single-cell transcriptomics analysis

scRNA-seq data from newly diagnosed GBM tumor tissues analyzed in this study include four datasets: LeBlanc et al.^29^ (10 patients, GSE173280), Abdelfattah et al.^30^ (10 patients, GSE182109), Nomura et al.^31^ (56 patients, GSE274546) and an in-house dataset from Ebert et al.^15^ (3 patients). Each dataset was individually preprocessed using the standard workflow in the Seurat package (Seurat v5) (https://satijalab.org/seurat/articles/pbmc3k_tutorial). This included quality control, log normalization (scale factor 10,000), data scaling, linear dimensional reduction, cell clustering and uniform manifold approximation and projection (UMAP) reduction. We used cell type annotations for the clusters as reported by the authors in the publications first reporting the generation of each dataset (GSE173280^29^, GSE182109^30^ and GSE274546^31^, Ebert et al.^15^). High relative expression of established pericyte markers (PDGFRB, THY1, ACTA2 and CSPG4) was used to confirm that pericyte clusters were annotated appropriately. Pericyte clusters from each dataset were subset and reclustered to resolve pericyte subpopulations. Highly variable genes (1000 features) were identified using variance-stabilizing transformation, followed by principal component analysis (PCA). The first 20 principal components were used to construct a shared nearest neighbor graph and perform graph-based clustering (resolution 0.1–0.3), with dimensionality selection guided by elbow plots. A UMAP was performed using 10–35 principal components, selected per dataset to optimize subcluster separation. We further identified specific marker genes for pericyte subpopulations using the ‘FindAllMarkers’ function (minimum percentage of ≥25% of cells in at least one group). The list of pericyte subpopulation markers was further refined based on cluster specificity across the four datasets. The resulting pericyte subpopulation markers (Supplementary Table 2) were then used to annotate pericyte cells in each dataset as follows: pericyte subpopulations for a total number of cells ${\boldsymbol{n}}$ and genes m having normalized expression E, were annotated by first calculating the scaled gene expression (${S}_{ij}$) for gene i in cell j as

$${S}_{{ij}}=\,{E}_{{ij}}/\left(\frac{{\sum }_{j=1}^{n}{E}_{{ij}}}{n}\right)\,.$$

From this, we then calculated the subpopulation score for the cell ${\boldsymbol{j}}$ over l genes in a gene list g as

$${N}_{{gj}}=\frac{{\sum }_{i\in g}{Sij}}{l}\left/\frac{{\sum }_{i\notin g}{Sij}}{m-l}\right.$$

Each cell is assigned the pericyte subpopulation for which it has a maximum score ${N}_{{gj}}$ greater than 1. Cells with a score less than 1 are not assigned to any subpopulation. To reduce cell-to-cell noise and increase robustness of pericyte annotations, pericyte subpopulation signature scores were smoothed using *k*-nearest neighbor averaging with the UCell SmoothKNN function. Pericyte subtype annotations were validated against results from an independent single-cell analysis of mural cell populations across different brain tumor disease cohorts including IDH-wild-type GBM^32^.

Pericyte subpopulation composition was quantified by calculating, within each dataset, the percentage of pericytes in each subpopulation for each patient and then averaging these patient-level percentages to obtain dataset-level means (rescaled to sum to 100%). Similarly, for FAP^+^ GBM cell type composition, FAP^+^ cells (FAP counts >0) were identified, and per-patient cell type percentages were quantified and averaged within each dataset (rescaled to sum to 100%).

The expression of marker genes was quantified for each pericyte subpopulation in each dataset. To facilitate comparison between datasets, gene counts for genes of interest were *z*-score normalized within each dataset. We identified DEGs in each dataset (log_2_FC >0.5 or <−0.5, and adjusted *P* value <0.05 as determined by Benjamini–Hochberg false discovery rate (FDR) analysis) between FAP^+^ (FAP counts >0) and FAP^−^ pericytes (FAP counts equal to 0) using the Seurat function ‘FindMarkers’. Overlapping the significantly up- and downregulated gene lists across the four datasets identified common DEGs. We use the ligand–receptor interaction database in CellChat^33^ to identify ligands of interest from the common DEG list. The identified ligand of interest was selected and quantified as *z*-score expression and percentage expressing (percent of cells with counts >0) across GBM cell types in each dataset. Analyses were performed both in FAP^+^ (FAP counts >0) across all cell type clusters and in the full, unfiltered cell population. Gene expression of the corresponding receptors was also quantified in myeloid subpopulations. These myeloid subpopulations were obtained by selecting the ‘myeloid’ cluster as defined by the dataset’s authors. Previously published lists of markers for myeloid subpopulations^34^ were used to annotate monocytes, macrophages and microglia using the same signature scoring method used for pericyte subpopulations. The macrophage population was then selected and further annotated as M1 or M2 using previously published markers^35^.

### Spatial transcriptomics analysis

Visium spatial transcriptomics data of newly diagnosed GBM tumor tissues in this study include Ravi et al.^36^ (20 patients) and Greenwald et al.^37^ (GSE237183, 13 samples from 6 patients).

For the Ravi dataset, data were downloaded as SPATA2 objects and converted to Seurat objects using the SPATA2 (v2.0.4) function, ‘asSeurat()’. The Spaceranger output was downloaded and used to create the Seurat objects for the Greenwald dataset. The data were individually processed following the standard spatial transcriptomics workflow in the Seurat package (Seurat v5) (https://satijalab.org/seurat/articles/spatial_vignette). Libraries were scaled to 10,000 UMIs per spot and log-normalized with the Seurat function ‘NormalizeData’. To analyze the spatial distribution of cell types in GBM, we used robust cell type decomposition (RCTD)^38^ analysis using the spacexr package (v2.2.0) to infer cellular composition in spatial transcriptomics data. We used the annotated Abdelfattah scRNA-seq dataset as a reference to identify cell types and subpopulations in the TME. This approach was used for broad cell types (for example, pericytes, endothelial cells, myeloid cells, malignant cells, lymphocytes and oligodendrocytes) as well as their corresponding subpopulations, including pericyte subpopulations (for example, FAP^+^ pericytes) and myeloid subpopulations. In addition, we annotated the malignant cell cluster in the scRNA-seq data using the GBM cell-state signatures described by Neftel et al.^39^ (NPC1, NPC2, OPC, AC, MES1 and MES2) and the same signature scoring method used for pericyte and myeloid subtype annotations. To facilitate broader downstream comparisons, related malignant states were subsequently consolidated into broader categories, with NPC1 and NPC2 grouped as NPC, and MES1 and MES2 grouped as MES. This analysis generated a cell type ‘proportion’ composition score (0–1) for each cell type per spot.

To identify tissues enriched in vessels, we summed the endothelial cell and pericyte composition scores to define a ‘vessel’ score. Spots with a vessel score ≥ mean + 1 standard deviation across all spots in the tissue were classified as ‘vessel enriched’. To ensure these enriched spots formed contiguous vessel structures, we computed a spot-aggregation score, that measured the percentage of surrounding ‘neighborhood’ spots (in Visium data, up to six neighbors) that were also vessel enriched. Only tissues with an average vessel aggregation score >0.3 were classified as vessel-enriched and included in further analysis (13/33 tissues). FAP^+^ pericyte-enriched spots were defined as those with a FAP^+^ pericyte composition score ≥ mean + 1 s.d. across all spots in the tissue. To enable comparison, vessel structures depleted of FAP^+^ pericytes were annotated as spots with a vessel score ≥ the vessel enrichment threshold and a FAP^+^ pericyte score < FAP^+^ pericyte enrichment threshold. log_2_FC differences in ligand expression were quantified in FAP^+^ pericyte-enriched spots versus FAP^+^ pericyte-depleted vessel spots per tissue. Significance was assessed by paired *t*-test and Benjamini–Hochberg multiple testing correction with *P* < 0.05 considered significant.

To estimate the relative contribution of ligand expression from different cell subtypes within FAP^+^ pericyte-enriched spatial regions, mean cell-type proportions (composition scores) were first quantified in FAP^+^ pericyte-enriched spots for each vessel-enriched tissue. Expression of ligands of interest was then assessed across four scRNA-seq datasets. For each dataset, average expression of each ligand was calculated within each annotated cell subtype and normalized to the global mean expression of that ligand across all cells within that dataset. This generated a dataset-specific normalized ligand expression score for each ligand–cell type pair. Normalized ligand expression scores were then averaged across datasets to generate an overall mean normalized expression value for each ligand–cell type pair. To account for the relative abundance of each cell subtype within FAP^+^ pericyte enriched spots, these mean normalized expression values were multiplied by the corresponding average cell-type proportion for each tissue within FAP^+^ pericyte enriched spots. This produced a contribution-weighted ligand expression score for each ligand–cell subtype combination in each tissue. For statistical comparisons, contribution-weighted expression scores were analyzed using a one-way analysis of variance (ANOVA) with cell subtype as the grouping variable. Ligand–cell subtype combinations were considered significant where Tukey-adjusted *P* values <0.001. For heat map visualization, mean normalized expression values were multiplied by the average cell-type proportion within FAP^+^ pericyte enriched spots across all tissues.

To measure log_2_FC differences in expression of myeloid markers in local neighborhoods surrounding FAP^+^ pericyte-enriched versus FAP^+^ pericyte-depleted vessel spots, the region was expanded to include the immediate six neighboring spots (seven-spot local neighborhood). Significance was once again assessed by *t*-test, with *P *< 0.05 considered significant.

To quantify the spatial enrichment of myeloid subtypes around FAP^+^ pericyte spots, we calculated the neighborhood composition score (NCS^40^). Spots with a FAP^+^ pericyte composition score ≥ the FAP^+^ pericyte enrichment threshold were identified, and the proportion of neighboring spots (within the seven-spot local neighborhood) enriched for each myeloid subtype (for example, M1/M2 macrophages, monocytes and microglia) was determined. The NCS was computed as the ratio of enriched myeloid subtype spots to total neighborhood spots, with average NCS values calculated per tissue. This analysis was also performed for other pericyte subpopulations annotated using RCTD analysis.

To observe how this spatial enrichment changes across regions proximal and distal to FAP^+^ pericyte-enriched spots, we quantified myeloid composition scores at increasing distances from these spots. Spots with a FAP^+^ pericyte composition score ≥ FAP^+^ pericyte enrichment threshold were identified, and the average composition score of each myeloid subtype was quantified per binned distance (100 µm). To facilitate cross-tissue comparison, average composition scores were normalized to 0–1. To enable direct comparison between FAP^+^ pericyte-enriched spots and other pericyte spots not enriched with FAP^+^ pericyte, we performed the same analysis around FAP^−^ pericyte spots defined as pericyte enriched spots with pericyte composition score ≥ pericyte enrichment threshold and FAP^+^ pericytes composition score < FAP^+^ pericyte enrichment threshold. This analysis was also performed for myeloid marker genes.

### Derivation of primary cell cultures and cultivation of cell lines

FAP^+^ pericyte-like cells were derived from fresh human GBM tissue according to a previously described procedure^19^. In brief, tumor tissue was dissociated mechanically and enzymatically (TrypLE, Gibco) and cultured in complete pericyte media, consisting of pericyte medium (PM) supplemented with 2% fetal bovine serum, pericyte growth supplement, 100 units/ml penicillin G and 100 µg/ml streptomycin (all provided by ScienCell Research Laboratories) on fibronectin-coated (2 µg/cm^2^, Merck) plastic. After 7 days, the FAP-expressing cells were isolated by magnetic-activated cell sorting using anti-FAP antibody (F11)-coated DynaBeads (Invitrogen). Cells were cultured on fibronectin-coated plastic under standard conditions at 37 °C in a humid atmosphere of 5% CO_2_ in a complete pericyte medium. Patient-derived GBM stem-like cell cultures (GSCs) were isolated as described previously^41^ and cultured in complete GSC medium consisting of DMEM/F-12 (Sigma-Aldrich) with 1% GlutaMAX (Thermo Fisher Scientific), 2% B-27 supplement minus vitamin A (Thermo Fisher Scientific), 100 U/ml penicillin, 100 μg/ml streptomycin (Sigma-Aldrich), 20 ng/ml EGF and 20 ng/ml FGF2 (PeproTech EC) in nonadherent cell culture flasks. The GSC cell line NCH644 (Cell Lines Service) was cultured in complete stem cell medium as described above. Cell cultures were tested for mycoplasma using the MycoAlert PLUS Mycoplasma Detection Kit (Lonza, LT07-703) and authenticated by short tandem repeat profiling performed by Generi Biotech.

Monocytes were isolated from healthy donors’ buffy coats by density gradient centrifugation using Ficoll-Paque (Cytiva) and by indirect magnetic-activated cell sorting using a Pan Monocyte Isolation Kit (Miltenyi Biotec) according to the manufacturer’s instructions. THP-1 monocytic cell line was a kind gift from Professor Klener (Institute of Pathological Physiology, First Faculty of Medicine, Charles University). Monocytes and THP-1 cells were cultured in RPMI1640 medium supplemented with 10% heat-inactivated fetal bovine serum (monocyte cultivation medium). The purity of isolated monocytes was verified by flow cytometric assessment of CD14 expression.

Primary mouse pericytes were isolated from adult C57BL/6 mice according to an established protocol^42^. In brief, six mouse brains were dissociated using the Papain dissociation system (Worthington Biochemical). Cells were initially cultured in endothelial-optimized conditions; after two passages, the medium was replaced with complete pericyte medium. To ensure culture purity, cells were characterized at each passage for the expression of pericyte markers: NG2, αSMA and contaminant markers: endothelial marker CD31, astrocyte marker GFAP and immune cell marker CD45. At the fourth passage, cells were positive for NG2 and α-SMA and negative for CD31, GFAP and CD45. Based on this marker profile, the cells were considered pericytes and were used for downstream in vivo experiment.

GL261 murine GBM cell line was acquired from NCI tumor repository and cultured in RPMI1640 medium supplemented with 10% of fetal bovine serum.

### In vivo co-implantation model

All animal experiments were approved by the Commission for Animal Welfare of the First Faculty of Medicine, Charles University, and by the Ministry of Education, Youth and Sports of the Czech Republic (MSMT-3828/2024-5), in accordance with national animal protection legislation. Eight-week-old male C57BL/6J mice (The Jackson Laboratory) were used for intracranial implantation. Surgical procedures were performed under intramuscular anesthesia with ketamine (100 mg/kg) and xylazine (10 mg/kg) to minimize animal suffering. Orthotopic tumors were generated using a stereotactic frame (Stoelting) by co-implantation of 1.7 × 10^4^ pericytes with 5 × 10^4^ GL261 GBM cells in a total volume of 4 µl (*n *= 7). Control mice received 5 × 10^4^ GL261 cells alone in a total volume of 4 µl (*n *= 7). Cells were injected at coordinates 1.2 mm anterior to bregma, 2.5 mm lateral to the midline and 3 mm deep into the right hemisphere at a rate of 1 µl/min using a Pump 11 Elite syringe pump (Harvard Apparatus). Mice were monitored and weighed daily. Brain tumors were collected 15 days post implantation, when the first mice in the cohort developed neurological symptoms or 10% body weight loss. Brains were embedded in Tissue Freezing Medium (Leica) and stored at −20 °C until further processing. Brains were sectioned coronally at a thickness of 10 µm using a cryotome (Leica). Sections were stained with hematoxylin and eosin for histological evaluation and volumetric analysis. From the first section showing detectable tumor tissue onward, every tenth section was collected for volumetric analysis. Tumor volume was analyzed using ImageJ software with the Volumest plugin.

### Immunocytochemistry

Primary FAP^+^ pericyte-like cell cultures and primary mouse pericytes growing on fibronectin-coated glass coverslips were fixed with 4% paraformaldehyde for 10 min at room temperature and permeabilized with 0.1% Triton X-100. Cells were incubated overnight at 4 °C with the following primary antibodies: anti-FAP (mouse hybridoma F19, ATCC, 55 µg/ml), anti-fibroblast (clone TE-7, Merck Millipore, 1:200), anti-NG2/MCSP (clone LHM 2, Novus Biologicals, 1:200), anti-αSMA (clone 1A7, Abcam, 1:100), anti-PDGFRβ (polyclonal, LS Bio, 1:100), anti-vWf (polyclonal, Dako, 1:200), anti-GFAP (clone GF-01, Exbio, 1:200) and anti-Sox2 (polyclonal, Abcam, 1:600), anti-FAP (clone AF3715, R&D Systems, 1:40), anti-NG2 (clone 546930, Invitrogen, 1:400), anti-CD31 (clone MEC 7.46, R&D Systems, 1:100), anti-GFAP (polyclonal, Abcam, 1:5000) and anti-CD45 (clone 30-F11, R&D Systems, 1:100). The primary antibodies were visualized using the corresponding secondary antibodies: anti-mouse Alexa Fluor 488 (Invitrogen), anti-rabbit Alexa Fluor 488 (Invitrogen), and nuclei were counterstained with Hoechst 33258 (Invitrogen, final concentration 0.5 ng/ml). Samples were mounted in Aqua-Poly/Mount (Polysciences). Images were acquired using an epifluorescence microscope (IX70, Olympus) with Orca-flash 4.0 camera (Hamamatsu).

### FISH

To verify the stromal origin of FAP^+^ pericyte-like cultures and rule out malignant cell contamination, fluorescence in situ hybridization (FISH) was performed using a dual-color probes (IntellMed) targeting the CDKN2A (9p21) locus (Orange) with the centromere 9 (CEN9) control (green) in cultures 95A and 109A, or the EGFR locus (orange) with the centromere 7 (CEN7) control (green) in cultures 69A and 90A. Cells growing on fibronectin-coated glass coverslips were fixed in a methanol and acetic acid solution (3:1), washed in 50 mM NH_4_Cl and permeabilized using 0.2% Triton X-100. Following dehydration through a graded ethanol series, the probes were hybridized for 5 min at 75 °C, followed by an overnight incubation at 37 °C in a humidified chamber. Remaining unbound probes were washed away using a washing solution I (0.4× saline-sodium citrate/0.3% NP-40) heated to 73 °C for 2 min and subsequently with washing solution II (2× saline-sodium citrate/0.1% NP-40) for 30 s at room temperature. Nuclei were counterstained with Hoechst 33258 (Invitrogen, final concentration 0.5 ng/ml). At least 100 nuclei per culture were analyzed by two independent observers using an Olympus fluorescence microscope. Images were acquired using an epifluorescence microscope (IX70, Olympus) with Orca-flash 4.0 camera (Hamamatsu). The status of the 9p deletion and EGFR amplification in the corresponding patient tumor tissue was established by the clinical pathology department during routine diagnostic examination.

### Preparation of conditioned media

To obtain serum-free conditioned media, 0.6 × 10^6^ FAP^+^ pericyte-like cells were seeded in 100-mm fibronectin-coated Petri dishes and grown in complete pericyte medium for 72 h. Then, the medium was removed, cells were washed twice with PBS, and 10 ml of serum-free RPMI 1640 medium or pericyte medium without supplements was added. Cells were cultured at 37 °C under standard conditions. After 72 h, the conditioned medium was collected, centrifuged (618*g* at 4 °C for 10 min), filtered through a 0.22-µm pore filter (Merck) and stored in aliquots at −80 °C. Corresponding media not exposed to cells were used as controls.

For secretome analysis, FAP^+^ pericyte-like cells and GSCs were seeded at 1 × 10^4^/cm^2^ in their corresponding complete media, grown for 72 h after which the media were replaced for fresh complete media. After additional 72 h, the conditioned media were collected and processed as described above.

### Analysis of conditioned media using xMAP Intelliflex DR-SE multiplex platform

Cytokine and chemokine levels in conditioned media from FAP^+^ pericyte-like cells and GSCs cultured in their corresponding complete medium were quantified using human cytokine/chemokine/growth factor magnetic bead panels A and B (Merck Life Science, HCYTPAB). Fluorescent signals were acquired on an xMAP Intelliflex DR-SE analyzer (version 2.1, Luminex Corporation), and analyte concentrations were calculated using the Belysa analysis software (version 1.2.2, Merck Life Science). All procedures were performed according to the manufacturer’s instructions.

### Monocyte migration

Monocyte migration was evaluated using a Transwell migration assay. For primary human monocytes, freshly isolated cells were resuspended in serum-free RPMI media and 2 × 10^5^ cells were seeded into the upper compartment of polycarbonate inserts with 5-µm pores (Corning). For THP-1 cells, 2 × 10⁵ cells were resuspended in serum-free pericyte medium and seeded into polycarbonate inserts with 8-µm pores (Falcon). In both assays, the lower compartment was filled with serum-free conditioned media from FAP^+^ pericyte-like cell cultures or the corresponding nonconditioned control media (serum-free RPMI for primary monocytes and pericyte medium without supplements for THP-1 cells). Recombinant CCL2 (100 ng/ml; Miltenyi) was used as a positive control. Cells were allowed to migrate for 2 h. For primary monocytes, nonmigrated cells were removed from the upper surface of the membrane using a cotton swab, and migrated cells were fixed with 4% paraformaldehyde, stained with crystal violet and manually counted. For THP-1 cells, transmigrated cells in the lower compartment were counted using an automated cell counter (Beckman Coulter). Primary monocyte migration assays were performed using monocytes from four different donors and conditioned media from four independent FAP^+^ pericyte-like cell cultures to account for potential donor-specific and cell culture-specific variations. Two inserts were used per condition, and migrating cells were quantified in five nonoverlapping visual fields per insert at 20× objective. THP-1 migration assays were repeated twice using conditioned media from six independent FAP⁺ pericyte-like cell cultures, with three inserts per condition.

### Monocyte polarization experiments, CSF1R inhibition

FAP^+^ pericyte-like cells (0.1 × 10^6^ per well) were seeded into six-well plates in complete pericyte medium. After 1 day, the medium was removed, cells were washed twice with PBS and 0.4 × 10^6^ freshly isolated monocytes in monocyte cultivation medium were added. For M1/M2 monocyte polarization, 1 × 10^6^ freshly isolated healthy donors’ monocytes per well were plated in a six-well plate in monocyte cultivation medium. To induce M1 macrophage polarization, cells were cultured with GM-CSF (50 ng/ml), and IFN-γ (50 ng/ml) and lipopolysaccharide (10 ng/ml) were added 24 h before collection. To induce M2 macrophage polarization, cells were cultured in M-CSF (50 ng/ml)-containing medium and IL-4 (20 ng/ml) was added 24 h before collection. Monocytes without treatment were cultured in complete monocyte cultivation medium. Media were changed on day 3 and day 5.

For CSF1R inhibition experiments, FAP^+^ pericyte-like cells (6 × 10^4^ cells/well) were seeded in the upper compartment of a Transwell insert (0.4-µm pore size, 24-well format; VWR). After 24 h, monocytes from healthy donors (*n *= 2; 2.4 × 10^5^ cells per well) were seeded in the lower compartment. The selective CSF1R inhibitor BLZ945 (MedChemExpress) was added to the medium (0.5 µM and 1 µM) at the onset of the 6-day differentiation period. Fresh medium containing BLZ945 was replenished on days 3 and 5. As positive controls, monocytes were concurrently differentiated into M1 and M2 macrophages over 6 days as described above.

On day 6, cells were collected using Accutase (Merck) and the expression of M1 (CD80, HLA-DR), and M2 (CD14, CD163 and CD206) polarization markers was quantified by flow cytometry.

### Flow cytometry

Flow cytometry was used to phenotype freshly isolated monocytes, macrophages generated in co-culture experiments, monocytes without treatment and classically (M1) or alternatively (M2) polarized macrophages. Cells were stained with fluorochrome-conjugated antibodies against CD14 (APC-Cy7, clone MEM-15, Exbio), CD45 (FITC, clone MEM-28, Exbio), HLA-DR (PE, clone L243, Sony), CD80 (APC, clone 2D10, Sony), CD206 (APC, clone 15-2, Sony) and CD163 (PE, clone GH1/61, Sony). Cell viability was assessed using Hoechst 33258 (Invitrogen, final concentration 5 µg/ml). Before antibody staining, cells were incubated with an Fc receptor-blocking solution (Miltenyi Biotec). Data acquisition was performed on a BD FACSVerse flow cytometer with the BD FACSuite software (Becton Dickinson), and data were analyzed using FlowJo software (TreeStar). For experiments involving CSF1R inhibition, a modified antibody panel was used consisting of HLA-DR (PE-CF594, clone L243, BD), CD80 (BV605, clone L307.4, BD), CD206 (BV421, clone 15-2, Sony) and CD163 (APC, clone GHI/61, Exbio). Cell viability was assessed using LIVE/DEAD Fixable Near-IR Dead Cell Stain Kit (Molecular Probes). Data acquisition for these experiments was performed using a NovoCyte Quanteon flow cytometer (Agilent Technologies), and data were analyzed using FlowJo software.

### Quantification of IFNγ production by T cells co-cultured with differentiated macrophages

Primary T cells were isolated from healthy donor buffy coats by negative magnetic selection (Pan T Cell Isolation Kit, Miltenyi Biotec). T cells were preactivated for 3 days using anti-CD3/CD2/CD28-coated beads (T Cell Activation/Expansion Kit, human; Miltenyi Biotec) according to the manufacturer’s recommendations. Monocytes (1 × 10⁵ cells/24WP well) were differentiated for 6 days under four conditions: (1) classically activated macrophages (M1: GM-CSF + IFNγ + LPS), (2) alternatively activated macrophages (M2: M-CSF + IL-4), (3) GSC-educated macrophages or (4) FAP⁺ pericyte-like cell-educated macrophages. GSCs or FAP⁺ pericyte-like cells (2.5 × 10⁴ cells) were seeded in the upper compartment of 24-well Transwell insert (0.4-µm pore size; VWR) 24 h before monocyte addition into the lower compartment to allow adherence. After 6 days of differentiation, Transwell inserts were removed, macrophages were thoroughly washed with prewarmed PBS, and activated T cells (5 × 10⁵ cells per well) were added.

After 72 h of co-culture, supernatants were collected, centrifuged to remove cellular debris and IFNγ concentrations were quantified using a human IFNγ Quantikine ELISA kit (R&D Systems) according to the manufacturer’s instructions. Absorbance was measured at 450 nm with wavelength correction at 570 nm using a microplate reader (Tecan). Cytokine concentrations were calculated by interpolation from a standard curve.

### Transcriptional signature of FAP^+^ pericyte-like cells and survival analysis

A transcriptional signature was derived from genes distinguishing FAP⁺ pericyte-like cells from other pericyte populations. To ensure specificity for FAP⁺ pericytes and minimize confounding from genes expressed by malignant, endothelial or other stromal cell types within the TME, genes with high expression in nonpericyte compartments (≥15% expressing cells and *z-*score ≥0 in more than 50% of dataset per cell type) were excluded. The refined ten-gene signature comprised FAP, LTBP2, CYP1B1, ASAM (CLMP), FBLN2, OGN, FMOD, BICC1, CXCL6 and SFRP2. Gene nomenclature was harmonized across datasets (ASAM/CLMP).

Survival analyses were performed using publicly available GBM cohorts, including TCGA RNA-seq (HiSeqV2), TCGA HT_HG-U133A microarray and CGGA mRNAseq_325 and mRNAseq_693 datasets. Only primary, IDH-wild-type GBMs with available overall survival data were included. Platform availability of signature genes was 10/10 in TCGA RNA-seq and both CGGA RNA-seq cohorts and 7/10 in the TCGA U133A microarray dataset (missing genes: CLMP/ASAM, BICC1 and SFRP2).

Within each cohort, gene expression values were analyzed independently. For RNA-seq datasets, expression values were log_2_-transformed where required and *z*-score-normalized per gene across samples within each cohort. A signature score was calculated as the mean *z*-score across the available signature genes in each cohort.

Overall survival was assessed using Cox proportional hazards regression, with the signature score modeled as both a continuous variable (trend analysis) and a categorical variable after stratifying patients into cohort-specific terciles. Survival differences between the upper and lower terciles were evaluated using Kaplan–Meier analysis with log-rank testing. All analyses were performed in R using the survival and survminer packages.

### Statistical analysis

Statistica 12 and GraphPad Prism 8.0 were used. Normality was assessed using the Shapiro–Wilk test. For datasets that did not meet the assumptions of normality, nonparametric tests were applied. When homogeneity of variances was not met, Welch’s correction was used. A two-sided *P *< 0.05 was considered statistically significant. A linear mixed model (ANOVA) was used to analyze data from monocyte migration experiments. The log_2_-transformed number of migrating monocytes was used as the dependent variable. For experiments using the THP-1 monocytic cell line, the chemoattractant type (control medium, conditioned media from individual FAP⁺ pericyte-like cell cultures and the positive control CCL2) was modeled as a fixed factor (treatment), and experimental repetition was included as a random factor. For experiments using primary human monocytes, the chemoattractant type was included as a fixed factor (factor A) in the model. Random factors included individual donors (factor B), the type of pericyte-like cell culture used to produce the conditioned medium (factor C), the interaction between these two factors (B × C) and individual inserts used in the modified Boyden chamber assay (factor D). PCA was used to evaluate the expression of M1 and M2 markers. In addition, paired *t*-tests were used for each marker to determine whether monocytes co-cultured with FAP^+^ pericyte-like cells more closely resembled the classically activated M1 macrophages or alternatively activated M2 macrophages. The null hypothesis posited that the co-cultured monocytes were as far from classically activated M1 macrophages as from alternatively activated M2 macrophages. Rejection of the null hypothesis was interpreted as resemblance to one of the above phenotypes.

## Results

### FAP expression is associated with higher immune cell infiltration in GBM

**Fig. 1:**
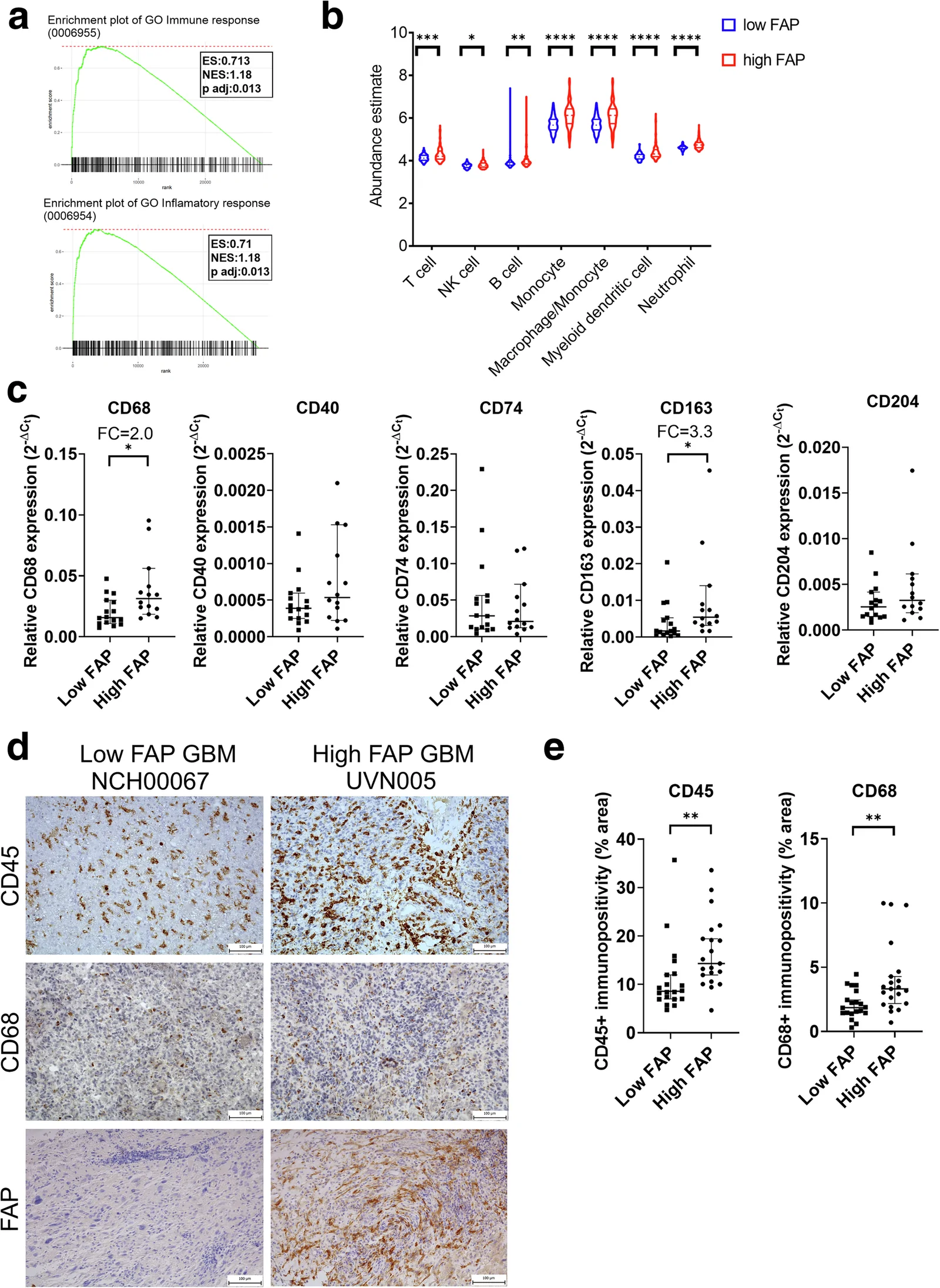
FAP expression is associated with proinflammatory state characterized by higher abundance of TAMs in GBM. **a** Gene set enrichment analysis (GSEA) of differentially regulated GO biological processes between FAP-high (*n *= 7) as compared with FAP-low (*n *= 6) GBMs. GSEA plots of the immune and inflammatory response are shown. **b** Quantification of immune cell subpopulations in FAP-high (*n *= 119, upper tercile) and FAP-low (*n *= 119, lower tercile) GBMs in TCGA dataset determined using the MCP counter, *P < 0.05, **P < 0.01, ***P < 0.001, ****P < 0.0001, two-way ANOVA multiple comparison test. **c** Relative expression of CD68, CD40, CD74, CD163 and CD204 in FAP-high (*n *= 14) and FAP-low (*n *= 16) GBMs determined by real-time qRT–PCR. **d** Representative immunohistochemistry images of CD45, CD68 and FAP in FAP-low and FAP-high GBMs. **e** Quantification of CD45^+^ and CD68^+^ cells in FAP-low and FAP-high GBMs as the percentage of immunopositive area. **P < 0.01, Mann–Whitney *U* test. The line is the median, and the whiskers are the 95% confidence interval. ES, enrichment score; FC, fold change; NES, normalized enrichment score.

In a pilot analysis, we categorized GBMs based on FAP expression determined by ELISA (*n *= 117 (refs. ^19,43^) and unpublished data) into FAP high (upper tercile, mean FAP expression 6.06 ng/mg of total protein, *n *= 39) and FAP low (lower tercile, mean FAP expression 1.2 ng/mg of total protein, *n *= 38). Transcriptome sequencing of seven FAP-high and six FAP-low GBMs revealed that gene sets comprised in GO biological processes related to immune response (GO0006955) and inflammatory response (GO0006954) were enriched in FAP-high GBM (Fig. 1a).

To interrogate the proportion of stromal cell types in GBM, we analyzed TCGA data from newly diagnosed IDH-wild-type GBMs using MCP Counter^21^ in the R immunedeconv package and stratified tumors into terciles based on FAP mRNA expression (FAP high, *n *= 119; FAP low, *n *= 119). As expected based on our previous studies^16,19^, nonmalignant mesenchymal and endothelial cells were significantly more abundant in the FAP-high GBM group (Supplementary Fig. 1a). In addition, abundance estimates of various subpopulations of lymphoid (T cells, NK cells and B cells) and myeloid cells (monocytes, macrophages, myeloid dendritic cells and neutrophils) were higher in the FAP-high GBM group, suggesting an immune-enriched TME (Fig. 1b). Consistent with these microenvironmental differences, the distribution of transcriptomic subtypes differed significantly between FAP-high and FAP-low GBMs (*χ*²(2) = 76.1, *P *< 0.0001; Cramér’s *V *= 0.56). FAP-high tumors were predominantly mesenchymal (60%), with smaller fractions of classical (31%) and proneural (9%) subtypes, whereas FAP-low tumors were mainly proneural (46%) or classical (45%), with only 9% classified as mesenchymal. NF1 alterations were more frequent in FAP-high tumors (12% versus 4%; *χ*²(1) = 4.73, *P *= 0.03; Fisher’s exact *P *= 0.033; odds ratio of 3.1, 95% CI 1.1–9.4; *Φ *= 0.14), consistent with the enrichment of the mesenchymal transcriptomic subtype. Conversely, PDGFRA alterations tended to occur more frequently in FAP-low tumors (16% versus 9%; *χ*²(1) = 3.11, *P *= 0.078; Fisher’s one-tailed *P *= 0.058; odds ratio of 2.06, 95% CI 0.91–4.64). Although not statistically significant, this trend mirrors the expected proneural enrichment of PDGFRA alterations and complements the inverse pattern observed for NF1 alterations.

We then focused in more detail on monocytes/macrophages, which represent up to 40% of cells in GBMs^44^. We analyzed the expression of monocyte/macrophage markers in 14 FAP-high and 16 FAP-low GBMs selected based on FAP protein concentration measured by ELISA. We tested ACTB, TUBB and GAPDH transcripts to normalize the target gene expression. ACTB exhibited the lowest variability and comparable expression in FAP-high and FAP-low tumors, and we therefore chose it for normalization. FAP transcript was more expressed in the FAP-high group (Supplementary Fig. 1b). The monocyte/macrophage lineage marker CD68 and M2 macrophage marker CD163 expression were twofold and threefold higher, respectively, in the FAP-high tumors. Differences in the expression of macrophage markers CD40, CD74 and CD204 between FAP-high and FAP-low GBMs did not reach statistical significance (Fig. 1c).

Previous studies have shown that FAP is predominantly expressed in pericyte-like cells in GBM^15,16^. We utilized samples from our previous study, where FAP expression was analyzed separately in stromal and cancer cells^19^. Tumors with more abundant FAP^+^ stroma (upper tercile, *n *= 21, mean stromal FAP score 2.26) exhibited higher immunopositivity of the leukocyte marker CD45 (16.48% ± 7.22% versus 10.99% ± 7.12% of tumor area) compared with tumors with low abundance of FAP^+^ stroma (lower tercile, *n *= 20, mean stromal FAP score 0.11; Fig. 1d, e). In addition, CD68^+^ macrophages were more abundant (4.07% ± 2.76% versus 2.10% ± 1.11% of tumor area) in tumors rich in FAP^+^ stroma (Fig. 1d, e and Supplementary Fig. 1c), confirming the association between macrophages and FAP^+^ pericyte-like cells in human GBMs.

### TAMs are in close contact with FAP^+^ pericyte-like cells in the perivascular niche

**Fig. 2:**
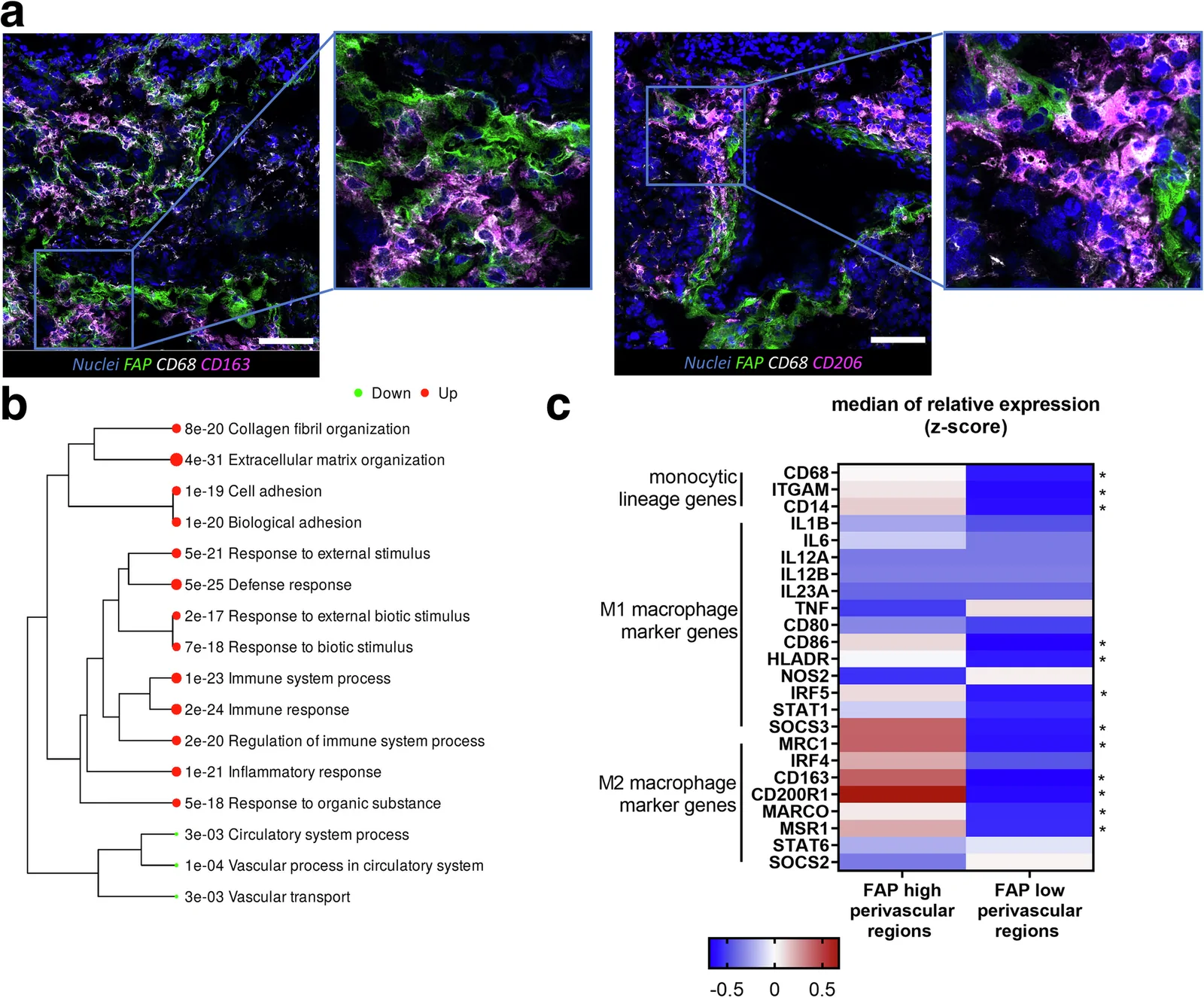
TAMs are in the proximity to FAP^+^ pericyte-like cells in GBM. **a** Macrophages expressing M2 markers CD163 and CD206 are localized in close proximity to FAP^+^ pericyte-like cells in GBM. Scale bar, 100 μm. **b** Enrichment analysis (GO biological processes) on DEGs in FAP-high versus FAP-low vascular regions in the Ivy Glioblastoma Atlas dataset. **c** Heat map depicting expression of monocyte/macrophage lineage markers and M1 and M2 macrophage phenotype markers in FAP-high versus FAP-low perivascular regions from the Ivy Glioblastoma Atlas dataset. *FDR <0.01, *t*-tests with FDR approach using two-stage step up method of Benjamini, Krieger and Yekutieli. ITGAM, integrin alpha M; HLA-DR, human leukocyte antigen DR; NOS2, inducible nitric oxide synthase; IRF5, interferon regulatory factor 5; IRF4, interferon regulatory factor 4; STAT1, signal transducer and activator of transcription 1; SOCS3, suppressor of cytokine signaling 3; CD200R1, CD200 receptor 1; MSR1, macrophage scavenger receptor 1 (CD204); MRC1, mannose receptor C-type 1 (CD206); MARCO, macrophage receptor with collagenous structure; STAT6, signal transducer and activator of transcription 6; SOCS2, suppressor of cytokine signaling 2.

To evaluate the spatial relations and possible interactions of FAP^+^ cells and monocytes/macrophages, we performed multicolor fluorescence immunostaining of GBMs (*n *= 5). Consistent with our previous findings^19^, FAP^+^ cells predominantly localized to perivascular regions (Fig. 2a), expressed mesenchymal markers (αSMA, PDGFRβ and NG2) and lacked expression of the glial marker GFAP (Supplementary Fig. 2).

Macrophages expressing M2 phenotypic markers (CD163 and CD206), typically associated with tumor-supporting functions, were frequently localized in the vicinity of FAP^+^ pericyte-like cells in GBM (Fig. 2a). To investigate further the possible association between FAP^+^ pericyte-like cells and monocytes/macrophages, we took advantage of the Ivy Glioblastoma Atlas dataset^18^. Samples from perivascular regions defined as hyperplastic blood vessels and microvascular proliferation, which display the highest FAP expression^15^, were divided into terciles based on the amount of FAP mRNA. FAP-high (upper tercile, *n* = 16) and FAP-low (lower tercile, *n* = 17) samples were subjected to analysis of DEGs and GO enrichment analysis using iDEP software. Among the significantly enriched GO biological processes, terms such as immune system processes, immune response, regulation of immune system processes and inflammatory response were identified (Fig. 2b).

Moreover, we found that FAP-high vascular tumor regions displayed statistically significantly higher expression of monocyte/macrophage lineage marker genes (CD68, CD14 and ITGAM/CD11b) (Fig. 2c) and genes related to macrophage activation and polarization (CD86, HLA-DR for M1-like and CD200R1, MRC1 (that is, CD206), MSR1 (that is, CD204), CD163 and MARCO for M2-like phenotype) in comparison to FAP-low perivascular regions (Fig. 2c). In addition, we observed higher expression of transcription factors associated with macrophage polarization toward M1-like (IRF5, SOCS3) and M2-like (IRF4) phenotypes.

These results suggest an association between FAP^+^ pericyte-like cells and the quantity of infiltrated monocytes and M1- and M2-like macrophage subpopulations in GBM perivascular regions.

### Single-cell and spatial transcriptomics reveal a distinct gene expression profile of FAP^+^ pericytes and their interaction with myeloid cells

**Fig. 3:**
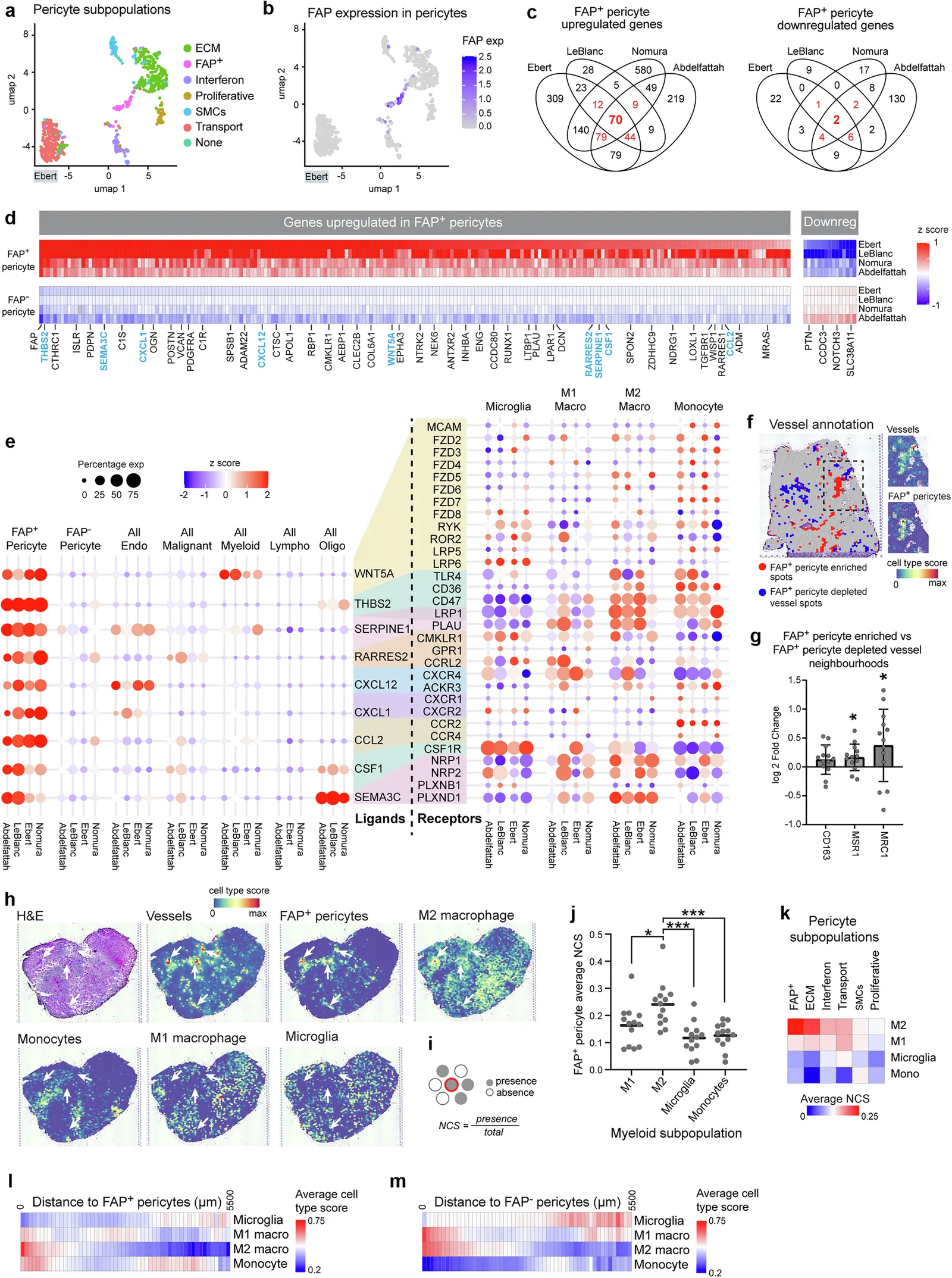
Single-cell and spatial transcriptomics analysis identifies a distinct FAP^+^ pericyte population localized to M2 macrophage-enriched hubs in the TME. **a** Pericyte subpopulations in GBM. Clustering analysis from four independent scRNA-seq datasets (Ebert^15^, Abdelfattah^30^, LeBlanc^29^ and Nomura^31^) comprising 79 GBMs identifies six conserved pericyte populations: ECM pericytes, FAP^+^ pericytes, interferon pericytes, proliferative pericytes, smooth muscle cells (SMCs) and transport pericytes. A representative UMAP from the Ebert dataset is shown (UMAPs for Abdelfattah and LeBlanc and Nomura, as well as a list of subpopulation markers are shown in Supplementary Fig. 4 and Supplementary Table 2). **b** FAP expression in the pericyte subpopulations in the Ebert dataset. **c** Venn diagrams of DEG expression analysis in FAP^+^ versus FAP^−^ pericytes. Overlap of DEGs across 4 datasets identifies 214 upregulated and 15 downregulated genes in FAP^+^ pericytes versus FAP^−^ pericytes in three out of four datasets (adjusted *P *< 0.05). **d** Heat map of FAP^+^ versus FAP^−^ pericytes DEGs. Genes significantly differentially expressed in three of four datasets were used to produce the heat map. Genes upregulated in FAP^+^ pericytes include many immune regulatory genes (highlighted in blue). **e** Dot plots showing the expression of key immune-regulatory ligands across FAP⁺ and FAP⁻ pericytes and other major cell populations (endothelial, malignant, myeloid, lymphoid and oligodendrocyte) in individual scRNA-seq datasets. Ligand expression is enriched in FAP⁺ pericytes compared with FAP⁻ pericytes and other major cell populations. Dot size indicates the percentage of expressing cells, and color intensity represents scaled expression (*z*-score). Cell type annotations were adopted from the original publications. Corresponding receptors show myeloid subtype dependent patterns of expression. Ligand expression in the FAP^+^ cell subpopulations of malignant, endothelial and myeloid cells, lymphocytes and oligodendrocytes is provided in Supplementary Fig. 5a. **f** Spatial transcriptomics plot showing the localization of FAP^+^ pericytes. Some vessels show overlap with FAP^+^ pericytes, whereas other vessel regions are FAP^+^ pericyte depleted. **g** Average log_2_ FC in expression of key myeloid subtype genes in FAP^+^ pericyte enriched neighborhoods versus FAP^+^ pericyte depleted vessel neighborhoods. For each annotated spot, the local neighborhood comprises the annotated spot and the six surrounding spots. Significant upregulation of the M2-associated genes *MSR1* (CD204) and *MRC1* (CD206) (*P *< 0.05) was observed in FAP^+^ pericyte enriched neighborhoods. Statistical significance was assessed by paired *t*-test and Benjamini–Hochberg multiple testing correction. *P < 0.05. **h** Spatial transcriptomics plots of nonmalignant cell populations from a representative tissue section. FAP^+^ pericytes localize to areas enriched in vessels and M2 macrophages. **i** NCS was calculated as the fraction of spots in which a given cell type is detected relative to the total number of spots within the local neighborhood. **j** Plot of the average NCS of each myeloid cell type in FAP^+^ pericyte neighborhoods. M2 macrophages are the most enriched myeloid subtype in FAP^+^ pericyte neighborhoods. Each datapoint represents the average NCS of an individual tissue; statistical significance was assessed using one-way ANOVA followed by Tukey post hoc test. *P < 0.05, ***P < 0.001. **k** Heat map of the average NCS of each myeloid subtype in each pericyte subpopulation neighborhood. FAP^+^ pericytes exhibit the highest NCS for M2 macrophages among all pericyte populations. **l**, **m** Heat maps illustrating the average distance (µm) of each myeloid population from FAP^+^ pericyte spots (**l**) and FAP^−^ pericyte spots (**m**). M2 macrophages are enriched in proximity to FAP^+^ pericytes compared with all other myeloid cells; however, monocytes are also more enriched close to FAP^+^ pericytes and depleted in regions close to FAP^−^ pericytes. All spatial transcriptomics analyses were performed on 13 vessel-enriched tissue sections from 10 patients with GBM obtained from two public datasets^36,37^.

Given the observed differences in myeloid cell composition between FAP^+^ and FAP^−^ vascular tumor regions, we next investigated transcriptional profiles of FAP^+^ and FAP^−^ pericytes using scRNA-seq data. We analyzed data from 79 patients with primary GBM from four datasets (Ebert et al.^15^, *n *= 3; Abdelfattah et al.^30^, *n* = 10; and LeBlanc et al.^29^, *n* = 10; Nomura et al.^31^, *n* = 56; 333,271 cells in total). These datasets contain malignant and various stromal cell populations (Supplementary Fig. 3a). Across datasets, FAP expression was detected in distinct cell populations, with variable FAP expression among cell types (Supplementary Fig. 3b) but higher expression in pericyte clusters, as identified by the dataset’s authors and confirmed by high expression of PDGFRB, THY1, ACTA2 and CSPG4 (Supplementary Fig. 3c). Subclustering of pericyte cell clusters revealed six distinct pericyte subpopulations in GBM (Fig. 3a and Supplementary Fig. 4a–c). The identity of these pericyte subclusters matched with recent annotations for different types of pericytes across brain tumor disease cohorts including IDH-wild-type GBM^32^ (Supplementary Fig. 4d). Notably, in all datasets, FAP^+^ pericytes formed a separate, transcriptionally distinct cell subpopulation (Fig. 3b and Supplementary Fig. 4a, c).

We then analyzed differential gene expression between FAP^+^ and FAP^−^ pericytes in these datasets. Compared with FAP^−^ pericytes, 214 genes were upregulated, whereas 15 genes were downregulated in FAP^+^ pericytes in at least three of the four datasets (Fig. 3c, d and Supplementary Table 3). Notably, various extracellular matrix (ECM) proteins including collagens (COL1A1, COL1A2, COL3A1, COL5A1, COL6A1, COL6A2, COL6A3, COL8A1, COL11A1, COL12A1 and COL13A1), fibulins (FBLN2 and FBLN5), proteoglycans (HSPG2, LUM, OGN and VCAN) and the glycoprotein EFEMP1, were consistently upregulated in FAP^+^ pericytes. In addition, genes involved in ECM synthesis (ADAMTS2 and SULF2) and turnover (PLAU, MMP2, MMP19, TIMP1 a nd LOXL1,2) were upregulated, suggesting an important role for FAP^+^ pericytes in ECM remodeling in GBM. These findings align with our recent proteomics analysis demonstrating the role of FAP^+^ pericytes in producing fibrillar ECM proteins in GBM^45^.

Interestingly, gene expression of several molecules involved in leukocyte recruitment was also upregulated in FAP^+^ pericytes, including CSF1, CCL2, CXCL1, CXCL12, RARRES2, SERPINE1, THBS2, WNT5A and SEMA3C (Fig. 3e). Notably, the chemokines CCL2 and CXCL1, as well as the matricellular protein THBS2, were expressed at markedly higher levels in FAP^+^ pericytes compared with malignant cells and other cells in the TME, including the FAP^+^ subpopulations of these cells, whereas CXCL12 and SERPINE1 were also expressed in endothelial cells and SEMA3C in oligodendrocytes (Fig. 3e and Supplementary Fig. 5a). CSF1, which encodes M-CSF (macrophage colony-stimulating factor), a cytokine involved in the proliferation, differentiation and survival of monocytes and macrophages, was upregulated in FAP^+^ pericytes in three of the four datasets. This finding further supports the potential role of FAP^+^ pericytes in modulating myeloid cells. Moreover, corresponding receptors for these ligands were expressed on myeloid cells, particularly in monocytes and M2-macrophages (TLR4, CD36, CD47, LRP1, CCR2 and PLXND1; Fig. 3e) and microglia and M2-macrophages (for example, CSF1R).

To further explore the spatial localization and relationships between these myeloid and pericyte cell subpopulations, we used RCTD^38^ to annotate FAP^+^ pericytes in spatial transcriptomics data (13 vessel-enriched tissue sections from 10 patients with GBM obtained from 2 public datasets^36,37^). Supporting their correct identification using the RCTD method, regions rich in FAP^+^ pericytes were predominantly localized around blood vessels and had higher expression of M2 markers, such as CD163, MSR1 and MRC1 in their neighborhoods, compared with vessel regions depleted of FAP^+^ pericytes (Fig. 3f, g). In addition, FAP⁺ pericyte-enriched regions exhibited significantly higher expression of multiple monocyte-recruiting and macrophage-polarizing ligands identified in the scRNA-seq analysis, including CCL2, CSF1, CXCL1, RARRES2 and THBS2 (Supplementary Fig. 6a). Because the local expression of the ligands within a tissue niche is influenced by both cell type-specific expression and cellular composition, we next integrated ligand expression data from the four independent scRNA-seq datasets with spatial cell-type proportions inferred by RCTD analysis. Although malignant cell populations represented the predominant cellular component of FAP⁺ pericyte-enriched regions, FAP⁺ pericytes showed disproportionately high contribution-weighted expression of several ligands, including CXCL1, THBS2 and WNT5A compared with most other cell populations within these regions. FAP⁺ pericytes were further among the major contributors to the expression of CCL2, CSF1 and RARRES2, together with malignant cell populations (Supplementary Fig. 6b, c). These findings suggest that FAP⁺ pericytes are not only enriched for the expression of ligands implicated in myeloid cell recruitment and polarization at the single-cell level but contribute substantially to the ligand-expression profile of FAP⁺ pericyte-enriched perivascular niches after accounting for cellular abundance. Using NCS analysis, we observed that M2 macrophages and, to a lesser extent, M1 macrophages, were more enriched in regions around FAP^+^ pericytes compared with monocytes and microglia (Fig. 3h, i, j). Of note, we found that this association was specific for FAP^+^ pericytes and, to a lesser extent, ECM pericytes but was absent in other pericyte subpopulations (Fig. 3k). This suggested M2 macrophages and FAP^+^ pericytes are close to each other in GBM, which we confirmed by analysis of distance distribution of M2 and M1 cell type scores (Fig. 3l) and gene marker expression (Supplementary Fig. 5b) from FAP^+^ and FAP^−^ pericytes. Interestingly, distance analysis revealed monocytes preferentially accumulate around FAP^+^ pericytes but not FAP^−^ pericytes (Fig. 3l, m).

In summary, these findings identify FAP^+^ pericytes as a distinct subpopulation characterized by ECM remodeling and expression of multiple ligands implicated in myeloid cell recruitment and polarization. Their preferential localization within vessel niches enriched for M2 macrophages, together with their substantial contribution to the ligand-expression profile of these niches, suggests that FAP^+^ pericytes may play an important role in shaping myeloid cell composition and phenotype in GBM.

### FAP^+^ pericyte-like cells induce monocyte chemotaxis

Single-cell transcriptomic analysis identified M-CSF (CSF1), SDF-1 (CXCL12) and CCL2 (MCP-1) as highly enriched transcripts in FAP^+^ pericytes in GBM. To validate these findings at the protein level and assess functional relevance, we isolated FAP^+^ pericyte-like cells from human GBMs as previously described^19^, prepared conditioned media and performed secretome analysis and monocyte Transwell migration assays.

Consistent with our previous data^19^, isolated FAP^+^ pericyte-like cell cultures expressed mesenchymal markers and lacked expression of markers associated with malignant, endothelial and hematopoietic cell populations (Supplementary Fig. 7). To further assess potential contamination with GBM cells, FISH targeting chromosome 9p deletion and EGFR amplification, and genomic alterations characteristic of GBM, was performed on representative FAP⁺ pericyte-like cultures derived using our established protocol. These analyses demonstrated absence of tumor-specific alterations, indicating that the isolation pipeline yields stromal cells that do not carry the GBM-specific genomic aberrations present in the corresponding tumor tissue (Supplementary Fig. 8).

**Fig. 4:**
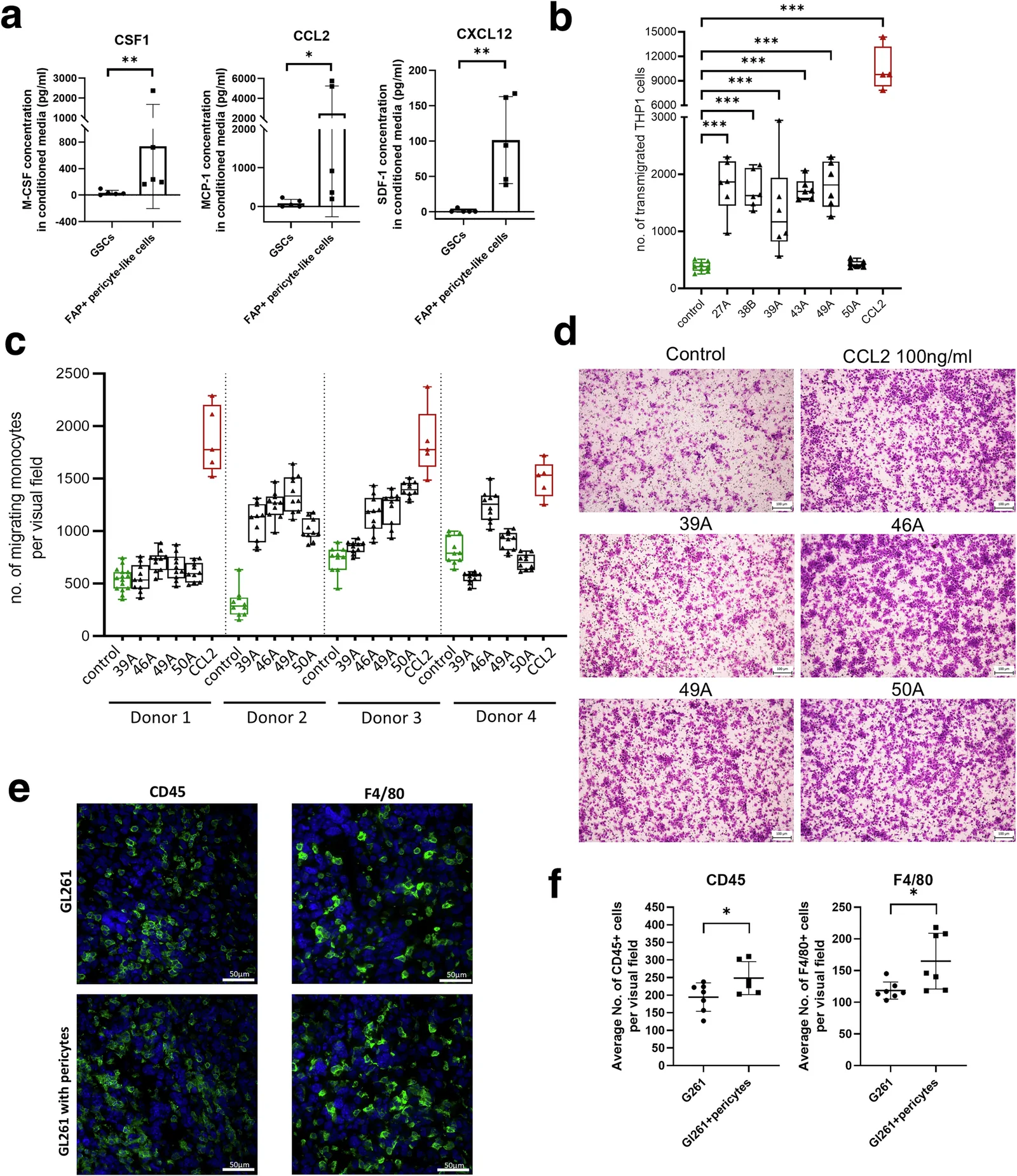
Monocytes are attracted to FAP^+^ pericyte-like cells isolated from human GBMs. **a** Protein levels of myeloid-modulating cytokines in conditioned media (CM) from FAP^+^ pericyte-like cells compared with patient-derived GSC cultures. Concentration (pg/ml) of CSF1, CXCL12 and CCL2 was determined using the xMAP Intelliflex DR-SE multiplex platform. *P < 0.05, **P < 0.01, Mann–Whitney *U* test. **b** Quantification of THP-1 monocyte migration in a Transwell assay. Conditioned media from individual FAP⁺ pericyte-like cell cultures (27A, 38B, 39A, 43A, 49A and 50A) were used as chemoattractants. Nonconditioned medium and 100 ng/ml CCL2 served as negative and positive controls, respectively. Each point represents the number of transmigrated cells in an individual well; data from two independent experiments. Conditioned media from five of six FAP⁺ cultures significantly increased migration compared with control medium, while one culture (50A) showed no significant effect. The line is the median, the box represents the interquartile range (25th to 75th percentile), the whiskers are the minimum to maximum values and the triangles are the individual wells; ***P < 0.001, linear mixed-effects model. **c** Quantification of transmigrating monocytes in a Transwell migration assay. Four independent experiments using monocytes isolated from four different healthy donors; conditioned media from four FAP^+^ pericyte-like cell cultures (39A, 46A, 49A, 50A) were used as a chemoattractant. Nonconditioned medium and 100 ng/ml CCL2 served as negative and positive controls, respectively. The line is the median, the box represents the interquartile range (25th to 75th percentile), the whiskers are the minimum to maximum values and the triangles are the individual wells; *P *< 0.001 for CCL2 versus control and *P *< 0.01 for FAP^+^ pericyte-like cell-conditioned media versus control, linear mixed-effects model. **d** Representative images of Transwell membranes with transmigrated monocytes stained with crystal violet. **e** Representative immunofluorescence images of CD45⁺ immune cells and F4/80⁺ macrophages in orthotopic GBMs generated by implantation of syngeneic GL261 cells alone or co-implanted with mouse brain-derived pericytes. Scale bar, 50 µm. **f** Quantification of CD45⁺ and F4/80⁺ cells per visual field in tumors derived from GL261 cells alone or co-implanted with pericytes. Each data point represents an individual mouse and corresponds to the mean of five independent high-power fields analyzed per tumor (*n *= 7 mice per group). Data are presented as mean ± s.d. *P < 0.05, unpaired two-tailed *t*-test.

Quantitative analysis of the conditioned media (CM) from FAP^+^ pericyte-like cells revealed significantly elevated levels of CSF1, CXCL12 and CCL2 compared with conditioned media from patient-derived GSC cultures, which represent a well-established in vitro model of GBM cells (Fig. 4a).

To functionally test the chemotactic potential of FAP^+^ pericyte-like cells, we first utilized THP-1 monocytic cells. Migration assays using CM from FAP^+^ pericyte-like cell cultures revealed significant increases in THP-1 monocyte migration in five of six cultures compared with control medium (linear mixed-effects model, *P *< 0.001), with fold increases ranging from 3.2- to 4.7-fold (Fig. 4b).

To further validate these findings using primary human cells, we used freshly isolated monocytes from healthy donor buffy coats. Monocytes were placed in the inserts and allowed to migrate toward FAP^+^ pericyte-like cell-conditioned media or CCL2 (100 ng/ml), a known monocyte chemoattractant, that we found upregulated in FAP^+^ pericyte-like cells (Figs. 3e and 4a). CCL2 induced a variable but reproducible increase in the number of migrating cells with a mean increase of 218% (*P *< 0.001, 95% confidence interval 91–430%, linear mixed model) compared with control nonconditioned media (Fig. 4c, d). Although the effect of FAP^+^ pericyte-like cell-conditioned media was more variable among individual donors, we observed a mean increase of 65% (95% confidence interval, 14–139%, linear mixed model) in the number of migrating cells compared with the negative control.

To determine whether these in vitro findings translate into enhanced myeloid recruitment within the TME in vivo, we assessed the impact of pericytes in an orthotopic GBM model. Selective depletion approaches were not feasible, as commonly used murine GBM models do not exhibit a robust and spatially organized FAP⁺ perivascular cell population comparable to that observed in human tumors. We therefore used an orthotopic co-implantation strategy to evaluate whether increasing pericyte abundance within tumors enhances immune cell infiltration in vivo.

Primary mouse brain pericytes were isolated (Supplementary Fig. 9a) and co-implanted intracranially with GL261 GBM cells into syngeneic immunocompetent mice. Mice were killed 15 days after implantation upon reaching predefined humane endpoints. Tumors co-implanted with pericytes exhibited a 27.7% increase in CD45⁺ cell infiltration compared with controls (mean difference 53.8 cells per visual field; 95% CI 0.6–106.9; two-tailed *t*-test, *P *= 0.0479). Similarly, F4/80⁺ macrophage density was increased by 39.4% in the pericyte-enriched tumors (mean difference 46.43, 95% CI 5.51–87.35; two-tailed Welch’s *t*-test, *P *= 0.0313) (Fig. 4e, f). Tumor volume was not significantly different between the two groups within the experimental timeframe (Supplementary Fig. 9b, c).

Together, these results demonstrate that FAP⁺ pericyte-like cells promote monocyte migration in vitro and pericyte enrichment in an orthotopic animal model is associated with increased myeloid infiltration.

### FAP^+^ pericyte-like cells induce monocyte-to-macrophage differentiation

To determine the effect of FAP^+^ pericyte-like cells on monocyte differentiation, we co-cultured them with healthy donor monocytes (*n *= 8 independent experiments) for 6 days. In parallel, monocytes were differentiated into classically (M1) and alternatively (M2) activated macrophages using GM-CSF + IFN-γ + lipopolysaccharide and M-CSF + IL-4, respectively. The expression of M1 (CD80, HLA-DR) and M2 (CD14, CD163 and CD206) polarization markers was analyzed using flow cytometry in CD45^+^ cells.

**Fig. 5:**
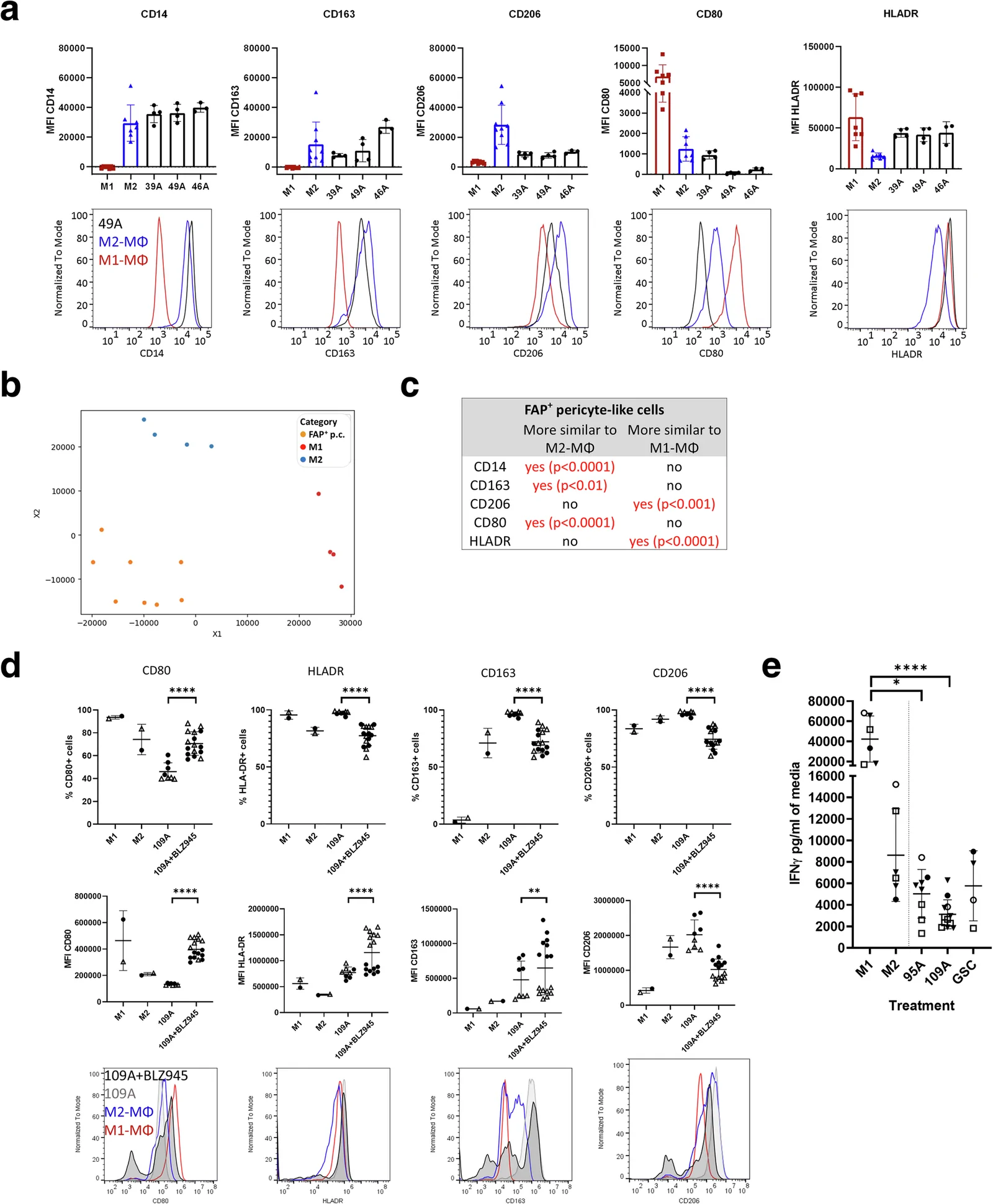
Co-culture of patient-derived FAP^+^ pericyte-like cells with healthy donor monocytes induces their differentiation into macrophages expressing M1 and M2 markers. **a** Expression of macrophage polarization markers determined by flow cytometry and representative histograms of monocytes co-cultured with FAP^+^ mesenchymal cultures (39A, 49A, 46A) or classically (M1) and alternatively (M2) polarized macrophages. **b** PCA of M1 (CD80 and HLA-DR) and M2 (CD14, CD163 and CD206) polarization marker expression (MFI) in monocytes co-cultured with FAP^+^ pericyte-like cells or classically (M1) and alternatively (M2) activated macrophages. **c** Results of paired *t*-tests evaluating whether monocytes co-cultured with FAP^+^ pericyte-like cells are more similar to M1 or M2 activated macrophages. **d** Flow cytometric analysis of macrophage phenotype in Transwell co-cultures following CSF1R inhibition. Percentage and MFI of CD206^+^, CD80^+^, HLA-DR^+^ and CD163^+^ macrophages following 6 days of co-culture with FAP^+^ pericyte-like cells in the presence of the CSF1R inhibitor BLZ945 (0.5 µM and 1 µM; data pooled) or control. Data represent individual replicates from two independent donors (open triangles and filled circles) with mean ± s.d. **P < 0.01, ****P < 0.001, main effect of treatment (two-way ANOVA). **e** A quantification of IFNγ production in T cell co-culture assays. Concentration of IFNγ (pg/ml) in supernatants of preactivated T cells co-cultured for 72 h with classically activated (M1), alternatively activated (M2), GSC-educated or FAP^+^ pericyte-like cell-educated macrophages. Data are presented as mean ± s.d. from five independent Transwell co-cultures derived from four donors. *P < 0.05, ****P < 0.001, Kruskal–Wallis ANOVA. MFI, mean fluorescence intensity.

Isolated primary monocytes cultured in medium alone for 6 days did not acquire macrophage morphology and showed limited viability, consistent with the requirement for exogenous survival signals. Consistent with expectations, classically (M1) activated macrophages predominantly upregulated CD80 and HLA-DR, while alternatively (M2) activated macrophages upregulated CD14, CD163 and CD206. Notably, expression of these markers was also observed in monocytes co-cultured with FAP^+^ pericyte-like cells (Fig. 5a and Supplementary Fig. 10). PCA showed that, based on the expression of polarization markers, monocytes co-cultured with FAP^+^ pericyte-like cells formed a cluster distinct from M1 and M2 activated macrophages (Fig. 5b).

We further used the MFI values of the M1 and M2 polarization markers in classically (M1) and alternatively (M2) activated macrophages as border values for the respective macrophage phenotypes and compared them with the MFI values in monocytes co-cultured with FAP^+^ pericyte-like cells using paired *t*-test. This allowed us to evaluate whether the co-cultured cells are more similar to M1 or M2-activated macrophages. In monocytes co-cultured with FAP^+^ pericyte-like cells, the expression of CD14, CD163 and CD80 was more similar to alternatively (M2) activated macrophages, whereas CD206 and HLA-DR were more similar to classically (M1) activated macrophages (Fig. 5c). Thus, cancer-associated FAP^+^ pericyte-like cells induce a mixed phenotype, expressing both M1 and M2 markers, a characteristic of TAMs. Building on our single-cell sequencing data and quantitative analysis of the conditioned media from FAP^+^ pericyte-like cells which both identified CSF1 as a signaling molecule secreted by FAP^+^ pericytes (Fig. 4a), we investigated whether the CSF1–CSF1R axis drives the observed monocyte-to-TAM transition. We co-cultured monocytes with FAP^+^ pericyte-like cells in the presence or absence of the selective CSF1 receptor (CSF1R) inhibitor BLZ945. Both inhibitor concentrations elicited comparable effects; therefore, the data were pooled for statistical analysis. Inhibition of CSF1R signaling significantly altered the resulting macrophage phenotype, characterized by a marked decrease in the percentage of CD206^+^ cells and a concomitant increase in CD80^+^ populations (Fig. 5d). Interestingly, the expression levels of HLA-DR remained largely unaffected, and for CD163, only the percentage of positive cells decreased with BLZ945 treatment, suggesting that additional FAP^+^ pericyte-derived factors may contribute to the mixed phenotype (Fig. 5d).

To determine if the TAM-like phenotype translates into functional immunosuppressive activity, we performed a T cell suppression assay. We co-cultured preactivated T cells (*n *= 4 independent donors) with FAP^+^ pericyte-like cell-educated macrophages and measured IFNγ production as a readout of T cell activation. Results were compared with those obtained from classically activated (M1-like), alternatively activated (M2-like) and GSC-educated macrophages. T cells co-cultured with macrophages educated by FAP⁺ pericyte-like cells exhibited IFN-γ production substantially lower than in co-cultures with M1-like macrophages and comparable to levels observed in co-cultures with M2-like and GSC-educated macrophages (Fig. 5e). These findings indicate that, despite displaying a mixed profile of M1 and M2 markers, FAP⁺ pericyte-like cell–educated macrophages exert an immunomodulatory effect functionally aligned with an M2-like state.

### A transcriptional signature of FAP^+^ pericyte-like cells is associated with worse survival in patients with GBM

**Fig. 6:**
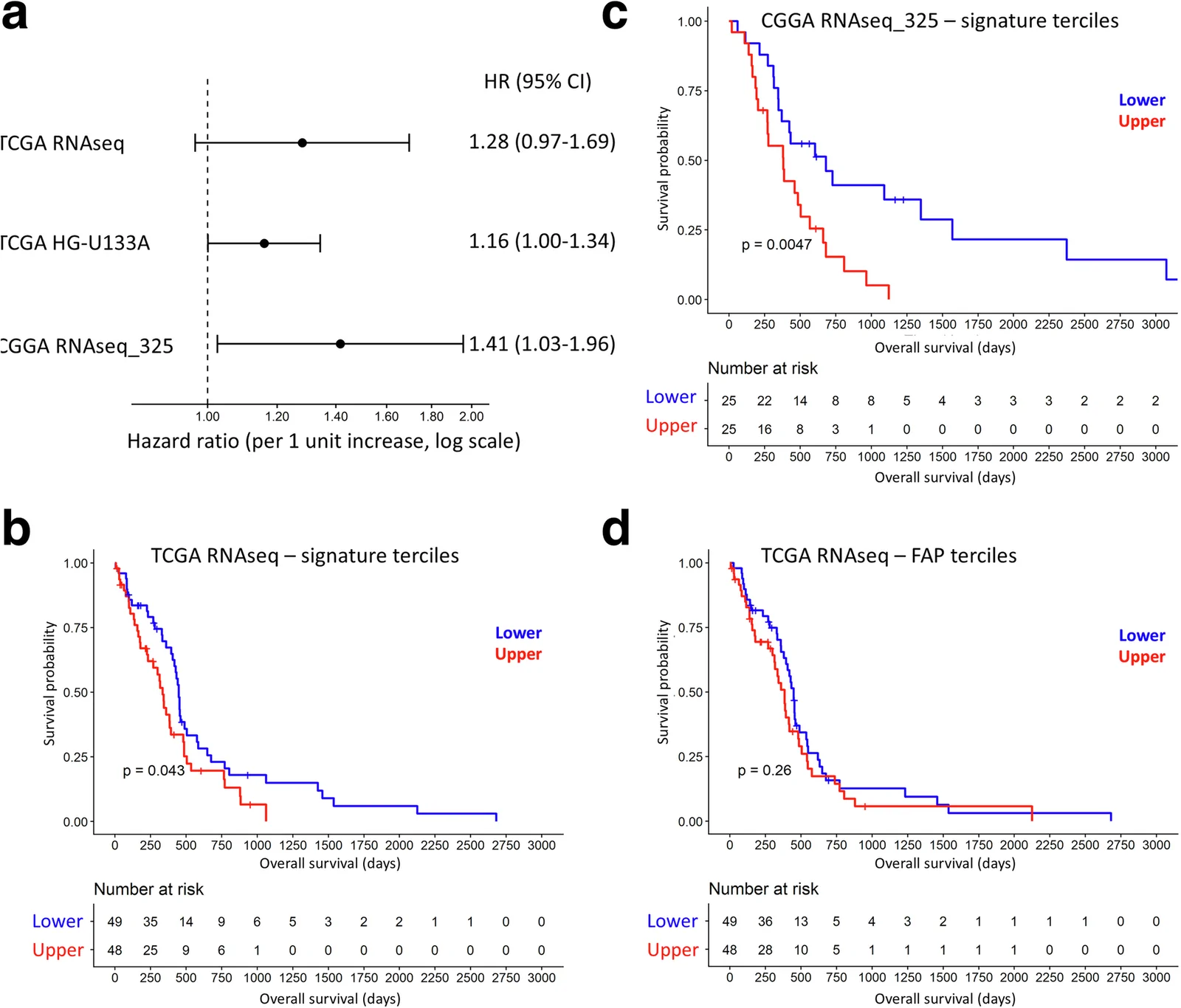
A transcriptional signature of FAP^+^ pericyte-like cells is associated with worse survival in IDH-wild-type GBM. **a** Forest plot showing HR estimates from Cox proportional hazards regression analyses evaluating the association between the FAP⁺ pericyte signature score (modeled as a continuous variable, per unit increase in the signature score) and overall survival in IDH-wild-type primary GBM cohorts: TCGA RNA-seq (*n *= 145), TCGA HG-U133A (*n *= 482) and CGGA RNAseq_325 (*n *= 74). Points represent HRs, horizontal lines indicate 95% confidence intervals and the dashed vertical line indicates HR of 1. **b** Kaplan–Meier plots for patients in the TCGA RNA-seq cohort stratified by terciles of the FAP⁺ pericyte signature score. Patients in the upper tercile exhibited significantly worse survival compared with those in the lower tercile (HR of 1.61, 95% CI 1.00–2.55, Cox proportional hazards model; log-rank *P *= 0.043). **c** Kaplan–Meier plots for patients in the independent CGGA mRNAseq_325 cohort stratified by terciles of the FAP⁺ pericyte signature score. Patients in the upper tercile showed significantly worse survival compared with those in the lower tercile (HR of 2.54, 95% CI 1.31–4.97, Cox proportional hazards model; log-rank *P *= 0.0047). **d** Kaplan–Meier plots for patients in the TCGA RNA-seq cohort stratified by terciles of FAP expression alone. FAP expression alone did not show a significant association with overall survival (log-rank *P *= 0.26).

To assess the clinical relevance of FAP⁺ pericyte-like cells, we first examined the association between FAP expression and survival. Although FAP expression has been associated with worse survival in studies examining the effect across gliomas of different grades^46^, its prognostic relevance in IDH-wild-type GBM remains inconsistent. In our reanalysis of TCGA and CGGA datasets restricted to IDH-wild-type primary GBM, FAP expression alone did not show a robust, reproducible association with overall survival (Fig. 6d and Supplementary Fig. 11).

Based on our single-cell and spatial transcriptomic analyses we constructed a ten-gene signature specific for FAP⁺ pericyte-like cells (Supplementary Fig. 12), excluding genes highly expressed by other cell types (Materials and methods).

We next evaluated the prognostic relevance of this signature in multiple independent IDH-wild-type primary GBM cohorts. Across datasets, higher signature expression was associated with worse overall survival, with strongest effects observed in the TCGA RNA-seq and CGGA mRNAseq_325 cohorts (Fig. 6 and Supplementary Table 4).

In the TCGA RNA-seq cohort (*n *= 145), patients in the upper tercile exhibited significantly increased hazard of death compared with the lowest tercile (hazard ratio (HR) of 1.61, 95% CI 1.00–2.55, *P *= 0.044), and an ordered trend analysis confirmed a dose–response relationship across increasing terciles (*P *= 0.034) (Fig. 6b).

These findings were supported in the expanded TCGA HG-U133A IDH-wild-type cohort (*n *= 482), where higher signature score was associated with worse OS in continuous Cox regression (HR of 1.16, *P *= 0.0489) (Fig. 6a). The effect remained directionally consistent after age adjustment (Supplementary Table 4).

Importantly, in the independent CGGA mRNAseq_325 cohort (*n *= 74), continuous Cox regression demonstrated a significant association between higher signature score and worse overall survival (HR of 1.42, 95% CI 1.03–1.96, *P *= 0.034). Patients with upper-tercile signature expression exhibited markedly worse survival compared with those with lower-tercile expression (HR of 2.54, 95% CI 1.31–4.97, *P *= 0.0061) (Fig. 6c).

By contrast, no significant association was observed in the CGGA mRNAseq_693 cohort (Supplementary Table 4). The estimated hazard ratios were below 1, indicating an inverse trend compared with the other cohorts and underscoring intercohort heterogeneity frequently reported in transcriptomic studies of GBM.

Collectively, these findings demonstrate that a transcriptional signature characteristic of FAP⁺ pericyte-like cells is associated with worse clinical outcome in IDH-wild-type GBM, supporting the translational relevance of this stromal cell population.

## Discussion

Bone marrow-derived monocytes represent an important source of tumor-promoting myeloid cells in GBM^44^. In this study, we identify FAP^+^ pericyte-like cells as a perivascular stromal population associated with monocyte recruitment and differentiation into TAMs. Tumors with high FAP expression exhibit an immune-enriched microenvironment characterized by increased abundance of myeloid and lymphoid cells. In particular, the abundance of macrophages and expression of M2 polarization markers are higher in FAP-high GBMs, and TAMs are frequently in close spatial proximity to FAP^+^ pericyte-like cells. Single-cell RNA sequencing and spatial transcriptomics further define FAP^+^ pericytes as a transcriptionally distinct stromal subpopulation enriched for ECM components and myeloid-modulating ligands. Consistent with this molecular profile, functional assays demonstrate that FAP^+^ pericyte-like cells isolated from human GBMs promote monocyte migration and differentiation into TAMs with M2-like characteristics, with evidence supporting a contributory role of the CSF1–CSF1R axis, and orthotopic co-implantation experiments in mice further support their capacity to enhance myeloid infiltration in vivo. Finally, a transcriptional signature characteristic of FAP^+^ pericytes correlates with worse overall survival in patients with GBM.

FAP, a cell surface marker of cancer-associated fibroblasts and pericytes, is upregulated in various tumor types, including GBMs, and represents a potential theranostic target^11^. In our study, FAP was used to stratify tumors according to the proportion of FAP^+^ pericyte-like cells. Although single-cell RNA sequencing data indicate that FAP transcripts are detectable in malignant cells, as previously reported^15^, the overall validity of this approach was supported by the higher number of nonmalignant mesenchymal stromal cells identified in FAP-high GBMs by the deconvolution algorithm. Abundance of endothelial cells was also higher in these tumors, suggesting enhanced vascularization, consistent with the previously reported proangiogenic function of the FAP^+^ pericyte-like cells^19^.

We complemented bioinformatic results with qRT–PCR analysis in tumors stratified by FAP protein level. Because the majority of GBMs do not show detectable FAP protein expression in malignant cells by immunohistochemistry^19^, protein-based stratification predominantly reflects stromal FAP expression. In this setting, both the pan-macrophage marker CD68 and the M2 macrophage marker CD163 were more expressed in FAP-high GBMs. This was further corroborated by immunohistochemistry analysis of GBM tissues, which showed that CD68^+^ macrophages were more frequent in GBMs with a high abundance of FAP^+^ stroma. In addition, using publicly available expression data from morphologically defined GBM regions and triple immunolabeling, we established that macrophages expressing CD163 and CD206 were in close proximity to FAP^+^ cells, suggesting their functional interplay. These observations are consistent with a recently published study based on the analysis of TCGA data, which showed that FAP expression positively correlated with the expression of M2 macrophage markers and macrophage abundance in GBM^47^.

Only a few studies addressed pericyte heterogeneity in gliomas. Supporting our findings, Guo et al. identified seven pericyte subpopulations, with one population possibly involved in immune cell infiltration and immunomodulation^48^. A recent preprint further highlighted the role of perivascular mesenchymal cells in recruiting TAMs^49^. Our analysis of four single-cell transcriptomic datasets (79 patients, 333,271 cells) revealed a set of genes consistently upregulated in FAP^+^ pericytes. Many of these genes encode ECM proteins, and several, including CSF1, CCL2, CXCL1, CXCL12, RARRES2, SERPINE1, THBS2, SEMA3C and WNT5A, are associated with leukocyte recruitment and differentiation. Specifically, CSF1, CCL2 and CXCL1 are known monocyte chemoattractants^50,51^. CXCL12 plays a role in the recruitment of monocytes and TAMs^52^, whereas RARRES2 and SEMA3C influence macrophage polarization^53,54^. SERPINE1 promotes the recruitment and polarization of monocytes into M2 macrophages^55^, THBS2 facilitates macrophage recruitment and their differentiation into TAMs^56^, and WNT5A induces M2 macrophage polarization and macrophage recruitment through CCL2 induction^57^. FAP^+^ pericytes highly expressed these ligands compared with malignant cells. Importantly, integration of single-cell and spatial transcriptomic data demonstrated that FAP⁺ pericyte-enriched regions are characterized by increased expression of several of these ligands. This was particularly evident for CXCL1, THBS2 and WNT5A, whereas FAP⁺ pericytes also contributed substantially to CCL2, CSF1 and RARRES2 expression together with malignant cells, which were abundant in these regions. Furthermore, our analysis confirmed the expression of the corresponding receptors in myeloid cells. Although it was not possible to stratify the samples used for spatial transcriptomics into FAP-high and FAP-low samples, the analysis revealed that M2 macrophages were localized closer to FAP^+^ pericytes compared with other myeloid subtypes.

We recently elucidated the role of FAP^+^ mesenchymal cells in angiogenesis, as well as in GBM cell migration and growth, effects that were mediated at least in part by soluble factors^19^. There is further an increasing appreciation that mesenchymal cells located within or close to the blood vessel wall, including pericytes, adventitial fibroblasts and mesenchymal stromal cells, play an important role in immunity and inflammation^58^. Studies in extracranial malignancies have shown that modulation of pericyte phenotype or function can alleviate immunosuppression, normalize tumor vasculature and enhance responses to immune checkpoint blockade^59–61^. Our findings extend this emerging concept into GBM.

We used FAP^+^ pericyte-like cell cultures derived from GBMs as established previously in our laboratory^19^ to test whether they affect monocyte migration and polarization in vitro. Conditioned media from these cultures contained higher levels of CSF1, CXCL12 and CCL2 than those from glioma cells, supporting the findings of transcriptomic analyses that FAP^+^ pericyte-like cells contribute substantially to the local production of these mediators. Migration of THP-1 cells increased 3.2–4.7-fold in response to five of six conditioned media. These results were further supported by migration assays using human blood monocytes from healthy donors. The promigratory effect of FAP^+^ pericyte-like cells, mediated by soluble factors in their conditioned media, was modest, with substantial variability in the chemotactic response among individual donors. This aligns with previous reports showing heterogeneous response of healthy donor monocytes to chemoattractants and differentiation stimuli^62,63^. CCL2, a potent and well-established chemoattractant for human blood monocytes, elicited robust migratory responses; however, the magnitude of the response also varied among donors (1.8-3.5-fold increase) at concentrations previously reported in the literature^50,64^. This interdonor variability, observed even with a defined and highly potent stimulus, highlights the intrinsic heterogeneity in migratory responsiveness of primary human monocytes. The underlying cause of this variability remains unclear, but it likely involves differential expression of chemokine receptors or other donor-specific biological factors.

To extend our in vitro observations, we used an orthotopic co-implantation model in which primary murine brain pericytes were implanted together with GL261 glioma cells. In this setting, pericyte enrichment was associated with increased infiltration of CD45⁺ leukocytes and F4/80⁺ macrophages, whereas tumor size was not significantly affected. Given the rapidly progressive nature of the GL261 model, changes in immune composition may not readily translate into measurable differences in tumor growth. It should be noted that this approach does not establish that implanted murine pericytes fully recapitulate the molecular phenotype of human FAP⁺ pericyte-like cells nor does it address long-term pericyte persistence in vivo. We therefore interpret these findings as supportive evidence that increasing pericyte abundance within a glioma-conditioned microenvironment is sufficient to modulate myeloid infiltration, rather than as a definitive mechanistic dissection of FAP⁺ pericyte function. Although several previous reports showed rather pro-tumorigenic functions of GBM-associated pericytes^19,65^, a recent work reported that genetic pericyte depletion in glioma models accelerated tumor progression, increased immune-suppressive macrophage polarization, and promoted mesenchymal transformation of tumor cells^66^. In contrast to their depletion model, we used a pericyte co-implantation strategy and observed increased myeloid infiltration without measurable differences in tumor size within the experimental timeframe. Although the experimental strategies differ, both depletion and enrichment-based approaches indicate that altering pericyte abundance substantially impacts the immune microenvironment in GBM. These observations also emphasize the context-dependent and potentially subtype-specific roles of these perivascular stromal cells.

Previous studies have shown that most TAMs in GBM originate from peripheral blood monocytes and are preferentially recruited to perivascular areas^4,67^. Given their perivascular localization, FAP^+^ pericyte-like cells and the cytokines and ECM components they produce are among the first GBM microenvironmental components to encounter infiltrating monocytes and may play a crucial role in guiding their differentiation and polarization^61^. Although FAP⁺ pericytes represent a numerically minor population in single-cell sequencing datasets, which may underestimate vascular-associated stromal compartments, their functional impact is likely to depend more on their strategic perivascular localization than on their overall tumor-wide abundance. In our experiments, co-culture of monocytes with FAP^+^ pericyte-like cells resulted in increased expression of M2 polarization markers, as well as the M1 polarization marker HLA-DR. This finding is consistent with a previous study showing that TAMs in GBM co-express canonical M1 and M2 polarization markers^8^. Similarly, a recent study using GBM-derived CAFs, which may originate from pericytes^68^, showed that these cells can induce M2 polarization of macrophages^17^. The use of conditioned media in that study suggested that soluble factors mediated these effects. In our study, transcriptomic and secretome analyses identified CSF1 as a candidate mediator of the effect of FAP^+^ pericyte-like cells. This was functionally supported by pharmacologic inhibition of CSF1R using BLZ945. CSF1R inhibition significantly reduced the proportion of CD206⁺ macrophages and increased CD80⁺ populations, whereas HLA-DR expression remained largely unaffected and only the percentage of CD163⁺ cells decreased. These findings indicate that CSF1/CSF1R signaling contributes to macrophage polarization in this context; however, the incomplete reversal of the phenotype suggests that additional cytokines, such as IL-6 and IL-10^69^ may also participate in shaping the mixed macrophage state. Another mechanism contributing to macrophage polarization may be the ECM, particularly the fibrillar ECM proteins produced by FAP^+^ pericyte-like cells^45^. Indeed, ECM scaffolds have been shown to drive a hybrid M1-M2 macrophage phenotype^70^. The biological relevance of this transition is reflected in the functional state of the resulting macrophages. Activated T cells in the presence of FAP⁺ pericyte-educated macrophages produced lower levels of the antitumor cytokine IFNγ than in the presence of M1-polarized macrophages and comparable levels to those observed with M2-polarized and GSC-educated macrophages. Thus, despite partial expression of M1-associated markers such as HLA-DR, their functional profile more closely aligned with M2-like macrophages. Other mechanisms leading to M2 polarization in GBM include the production of CHI3L1 by glioma cells^71^, hypoxia in the GBM microenvironment^72^ and positive feedback mediated by soluble factors released by M2 macrophages^73^. Our findings suggest that FAP⁺ pericyte-like cells represent an additional perivascular stromal component capable of shaping local myeloid differentiation within this broader immunosuppressive network.

The direct involvement of the FAP molecule itself in the observed effects of FAP^+^ pericyte-like cells on monocytes remains unclear. A study using a glioma cell line endogenously expressing FAP revealed that conditioned medium from FAP-knockdown cells was less effective in promoting the M2 polarization in a human monocytic cell line U937, but the mechanism remains poorly defined^47^.

Elevated FAP expression in gliomas has been associated with adverse outcomes when analyzed across tumor grades^46^, yet its prognostic relevance specifically in IDH-wild-type GBM has been inconsistent and appears dependent on cohort composition and cut-off selection^15,16,19^. Because our single-cell and spatial transcriptomic analyses identified a distinct transcriptional program characteristic of FAP⁺ pericyte-like cells, we hypothesized that a gene signature reflecting this stromal cell population would more accurately capture its biological and clinical relevance than FAP expression alone. Indeed, higher expression of the FAP⁺ pericyte-like cell signature was associated with worse overall survival in independent IDH-wild-type GBM datasets.

These observations are consistent with prior studies linking increased pericyte abundance to resistance to temozolomide in GBM^74^ and demonstrating that tumor-associated pericyte gene programs are associated with progression and adverse outcome in low-grade gliomas^75^. By contrast, in the CGGA mRNAseq_693 cohort, we observed an inverse association, with hazard ratios below 1. Although all cohorts were restricted to primary IDH-wild-type GBM, differences in sequencing platform, cohort composition, treatment context or residual clinical heterogeneity may contribute to this discordance, underscoring the well-recognized variability of transcriptomic survival associations across independent GBM cohorts.

Collectively, these findings support the notion that FAP expression alone is unlikely to account for the observed biological and clinical associations; rather, FAP may mark a broader perivascular stromal program characterized by ECM remodeling and myeloid-modulating ligand expression. In this context, our study identifies FAP^+^ pericyte-like cells as a distinct stromal subpopulation in GBM associated with increased myeloid cell infiltration. Consistent with their enrichment in ECM components and myeloid-modulating ligands, these cells can stimulate monocyte migration and induce their differentiation into TAMs. We posit that FAP^+^ pericyte-like cells contribute to the immunosuppressive and tumor-supportive nature of the GBM microenvironment and may represent potential therapeutic targets.

## Supplementary information

Supplementary Information

Supplementary Table 3

## Data Availability

The bulk RNA-sequencing data generated in this study are available from the corresponding authors upon reasonable request. scRNA-seq data used in this study include previously published datasets from LeBlanc et al.^29^ (GSE173280), Abdelfattah et al.^30^ (GSE182109), Nomura et al.^31^ (GSE274546) and Ebert et al.^15^ (data available from authors upon request). Spatial transcriptomics data from 13 vessel-enriched tissue sections from 10 patients diagnosed with newly diagnosed GBM were obtained from datasets generated by Ravi et al.^36^ (available via Dryad at https://doi.org/10.5061/dryad.h70rxwdmj (ref. ^36^)) and Greenwald et al.^37^ (GSE237183). Codes for single-cell and spatial transcriptomics analysis associated with this project are available via GitHub at https://github.com/chloe-shard/Houdova_et_al_2026.

## References

[1] DeCordova, S. et al. Molecular heterogeneity and immunosuppressive microenvironment in glioblastoma. *Front. Immunol.* **11**, 1402 (2020). 10.3389/fimmu.2020.01402 PMC7379131 32765498

[2] Klemm, F. et al. Interrogation of the microenvironmental landscape in brain tumors reveals disease-specific alterations of immune cells. *Cell* **181**, 1643–1660 (2020). 10.1016/j.cell.2020.05.007 PMC8558904 32470396

[3] Friebel, E. et al. Single-cell mapping of human brain cancer reveals tumor-specific instruction of tissue-invading leukocytes. *Cell* **181**, 1626–1642 (2020). 10.1016/j.cell.2020.04.055 32470397

[4] Chen, A. X. et al. Single-cell characterization of macrophages in glioblastoma reveals MARCO as a mesenchymal pro-tumor marker. *Genome Med.* **13**, 1–13 (2021). 10.1186/s13073-021-00906-x PMC8136167 34011400

[5] Russo Dello, C. & Cappoli, N. Glioma associated microglia/macrophages, a potential pharmacological target to promote antitumor inflammatory immune response in the treatment of glioblastoma. *Neuroimmunol. Neuroinflamm.* **5**, 36 (2018).

[6] Ran, X. et al. Single-cell transcriptomics reveals the heterogeneity of the immune landscape of IDH–wild-type high-grade gliomas. *Cancer Immunol. Res.* **12**, 232–246 (2024). 10.1158/2326-6066.CIR-23-0211 PMC10835213 38091354

[7] Wang, X. et al. New insights from the single-cell level: tumor associated macrophages heterogeneity and personalized therapy. *Biomed. Pharmacother.* **153**, 113343 (2022). 10.1016/j.biopha.2022.113343 35785706

[8] Müller, S. et al. Single-cell profiling of human gliomas reveals macrophage ontogeny as a basis for regional differences in macrophage activation in the tumor microenvironment. *Genome Biol.* **18**, 1–14 (2017). 10.1186/s13059-017-1362-4 PMC5738907 29262845

[9] Sahai, E. et al. A framework for advancing our understanding of cancer-associated fibroblasts. *Nat. Rev. Cancer* **20**, 174–186 (2020). 10.1038/s41568-019-0238-1 PMC7046529 31980749

[10] Puré, E. & Blomberg, R. Pro-tumorigenic roles of fibroblast activation protein in cancer: back to the basics. *Oncogene* **37**, 4343–4357 (2018). 10.1038/s41388-018-0275-3 PMC6092565 29720723

[11] Busek, P., Mateu-Sanz, R., Zubal, M., Kotackova, L. & Sedo, A. Targeting fibroblast activation protein in cancer - Prospects and caveats. *Front. Biosci.* **23**, 1933–1968 (2018). 10.2741/4682 29772538

[12] Chen, X. & Song, E. Turning foes to friends: targeting cancer-associated fibroblasts. *Nat. Rev. Drug Discov.* **18**, 99–115 (2019). 10.1038/s41573-018-0004-1 30470818

[13] Barrett, R. & Puré, E. Cancer-associated fibroblasts: key determinants of tumor immunity and immunotherapy. *Curr. Opin. Immunol.* **64**, 80–87, 10.1016/j.coi.2020.03.004 (2020). PMC8228400 32402828

[14] Quail, D. F. & Joyce, J. A. The microenvironmental landscape of brain tumors. *Cancer Cell* **31**, 326–341 (2017). 10.1016/j.ccell.2017.02.009 PMC5424263 28292436

[15] Ebert, L. M. et al. Endothelial, pericyte and tumor cell expression in glioblastoma identifies fibroblast activation protein (FAP) as an excellent target for immunotherapy. *Clin. Transl. Immunol.* **9**, e1191 (2020). 10.1002/cti2.1191 PMC7557106 33082953

[16] Busek, P. et al. Fibroblast activation protein alpha is expressed by transformed and stromal cells and is associated with mesenchymal features in glioblastoma. *Tumour Biol.* **37**, 13961–13971 (2016). 10.1007/s13277-016-5274-9 27492457

[17] Jain, S. et al. Single-cell RNA sequencing and spatial transcriptomics reveal cancer-associated fibroblasts in glioblastoma with protumoral effects. *J. Clin. Invest*. **133**, e147087 (2023). 10.1172/JCI147087 PMC9974099 36856115

[18] Puchalski, R. B. et al. An anatomic transcriptional atlas of human glioblastoma. *Science* **360**, 660–663 (2018). 10.1126/science.aaf2666 PMC6414061 29748285

[19] Balaziova, E. et al. Fibroblast activation protein expressing mesenchymal cells promote glioblastoma angiogenesis. *Cancers* **13**, 3304 (2021). 10.3390/cancers13133304 PMC8267680 34282761

[20] Bowman, R. L., Wang, Q., Carro, A., Verhaak, R. G. W. & Squatrito, M. GlioVis data portal for visualization and analysis of brain tumor expression datasets. *Neuro. Oncol.* **19**, 139–141 (2017). 10.1093/neuonc/now247 PMC5193031 28031383

[21] Becht, E. et al. Estimating the population abundance of tissue-infiltrating immune and stromal cell populations using gene expression. *Genome Biol.* **17**, 218 (2016). 10.1186/s13059-016-1113-y PMC5134277 27908289

[22] Ewels, P. A. et al. The nf-core framework for community-curated bioinformatics pipelines. *Nat. Biotechnol.* **38**, 276–278 (2020). 10.1038/s41587-020-0439-x 32055031

[23] Kim, D., Langmead, B. & Salzberg, S. L. HISAT: a fast spliced aligner with low memory requirements. *Nat. Methods* **12**, 357–360 (2015). 10.1038/nmeth.3317 PMC4655817 25751142

[24] Liao, Y., Smyth, G. K. & Shi, W. featureCounts: an efficient general purpose program for assigning sequence reads to genomic features. *Bioinformatics* **30**, 923–930 (2014). 10.1093/bioinformatics/btt656 24227677

[25] Yates, A. D. et al. Ensembl 2020. *Nucleic Acids Res.* **48**, D682–D688 (2020). 10.1093/nar/gkz966 PMC7145704 31691826

[26] Ashburner, M. et al. Gene ontology: tool for the unification of biology. *Nat. Genet.* **25**, 25–29 (2000). 10.1038/75556 PMC3037419 10802651

[27] Korotkevich, G. et al. Fast gene set enrichment analysis. Preprint at *bioRxiv* 10.1101/060012 (2021).

[28] Abràmoff, M., Magalhães, P. J. & Ram, S. Image processing with ImageJ. *Biophotonics Int.* **11**, 36–42 (2004).

[29] LeBlanc, V. G. et al. Single-cell landscapes of primary glioblastomas and matched explants and cell lines show variable retention of inter- and intratumor heterogeneity. *Cancer Cell* **40**, 379–392 (2022). 10.1016/j.ccell.2022.02.016 35303420

[30] Abdelfattah, N. et al. Single-cell analysis of human glioma and immune cells identifies S100A4 as an immunotherapy target. *Nat. Commun*. **13**, 767 (2022). 10.1038/s41467-022-28372-y PMC8828877 35140215

[31] Nomura, M. et al. The multilayered transcriptional architecture of glioblastoma ecosystems. *Nat. Genet.* **57**, 1155–1167 (2025). 10.1038/s41588-025-02167-5 PMC12081307 40346361

[32] Bejarano, L. et al. Single-cell atlas of endothelial and mural cells across primary and metastatic brain tumors. *Immunity* **58**, 1015–1032 (2025). 10.1016/j.immuni.2025.02.022 40107274

[33] Jin, S. et al. Inference and analysis of cell-cell communication using CellChat. *Nat. Commun*. **12**, 1088 (2021). 10.1038/s41467-021-21246-9 PMC7889871 33597522

[34] Pombo Antunes, A. R. et al. Single-cell profiling of myeloid cells in glioblastoma across species and disease stage reveals macrophage competition and specialization. *Nat. Neurosci.* **24**, 595–610 (2021). 10.1038/s41593-020-00789-y 33782623

[35] Mantovani, A. et al. The chemokine system in diverse forms of macrophage activation and polarization. *Trends Immunol.* **25**, 677–686 (2004). 10.1016/j.it.2004.09.015 15530839

[36] Ravi, V. M. et al. Spatially resolved multi-omics deciphers bidirectional tumor-host interdependence in glioblastoma. *Cancer Cell* **40**, 639–655 (2022). 10.1016/j.ccell.2022.05.009 35700707

[37] Greenwald, A. C. et al. Integrative spatial analysis reveals a multi-layered organization of glioblastoma. *Cell* **187**, 2485–2501 (2024). 10.1016/j.cell.2024.03.029 PMC11088502 38653236

[38] Cable, D. M. et al. Robust decomposition of cell type mixtures in spatial transcriptomics. *Nat. Biotechnol.* **40**, 517–526 (2021). 10.1038/s41587-021-00830-w PMC8606190 33603203

[39] Neftel, C. et al. An integrative model of cellular states, plasticity, and genetics for glioblastoma. *Cell* **178**, 835–849 (2019). 10.1016/j.cell.2019.06.024 PMC6703186 31327527

[40] Barkley, D. et al. Cancer cell states recur across tumor types and form specific interactions with the tumor microenvironment. *Nat. Genet.* **54**, 1192 (2022). 10.1038/s41588-022-01141-9 PMC9886402 35931863

[41] Sana, J. et al. Identification of microRNAs differentially expressed in glioblastoma stem-like cells and their association with patient survival. *Sci. Rep.* **8**, 1–11 (2018). 10.1038/s41598-018-20929-6 PMC5809429 29434344

[42] Boroujerdi, A., Tigges, U., Welser-Alves, J. V. & Milner, R. Isolation and culture of primary pericytes from mouse brain. *Methods Mol. Biol.* **1135**, 383–392 (2014). 10.1007/978-1-4939-0320-7_31 24510880

[43] Krepela, E. et al. Regulation of fibroblast activation protein by transforming growth factor beta-1 in glioblastoma microenvironment. *Int. J. Mol. Sci.* **22**, 1–20 (2021). 10.3390/ijms22031046 PMC7864518 33494271

[44] Buonfiglioli, A. & Hambardzumyan, D. Macrophages and microglia: the cerberus of glioblastoma. *Acta Neuropathol. Commun.* **9**, 1–21 (2021). 10.1186/s40478-021-01156-z PMC7992800 33766119

[45] Vymola, P. et al. Fibrillar extracellular matrix produced by pericyte-like cells facilitates glioma cell dissemination. *Brain Pathol*. **34**, e13265 (2024). 10.1111/bpa.13265 PMC11483521 38705944

[46] Li, M. et al. Characterization and oncolytic virus targeting of FAP-expressing tumor-associated pericytes in glioblastoma. *Acta Neuropathol. Commun*. **8**, 221 (2020). 10.1186/s40478-020-01096-0 PMC7730751 33308315

[47] Miao, Y. et al. Anti-cancer effect of targeting fibroblast activation protein alpha in glioblastoma through remodeling macrophage phenotype and suppressing tumor progression. *CNS Neurosci. Ther.* **29**, 878 (2023). 10.1111/cns.14024 PMC9928553 36382346

[48] Guo, Y. et al. Single-cell transcriptomic analysis reveals the functional heterogeneity and clinical signification of pericyte subpopulations in gliomas. Preprint at *Research Square*10.21203/rs.3.rs-3023622/v1 (2023).

[49] Zarodniuk, M., Steele, A., Lu, X., Li, J. & Datta, M. CNS tumor stroma transcriptomics identify perivascular fibroblasts as predictors of immunotherapy resistance in glioblastoma patients. *NPJ Genom Med.* **8**, 35 (2023). 10.1038/s41525-023-00381-w PMC10603041 37884531

[50] Yoshimura, T. The chemokine MCP-1 (CCL2) in the host interaction with cancer: a foe or ally? *Cell*. *Mol. Immunol.* **15**, 335 (2018). 10.1038/cmi.2017.135 PMC6052833 29375123

[51] Hu, J. et al. Regulation of tumor immune suppression and cancer cell survival by CXCL1/2 elevation in glioblastoma multiforme. *Sci. Adv*. **7**, eabc2511 (2021). 10.1126/sciadv.abc2511 PMC7840139 33571109

[52] Zhou, X., Jin, G., Zhang, J. & Liu, F. Recruitment mechanisms and therapeutic implications of tumor-associated macrophages in the glioma microenvironment. *Front. Immunol.* **14**, 1067641 (2023). 10.3389/fimmu.2023.1067641 PMC10157099 37153567

[53] Qi, J. et al. Single-cell and spatial analysis reveal interaction of FAP+ fibroblasts and SPP1+ macrophages in colorectal cancer. *Nat. Commun.* **13**, 1–20 (2022). 10.1038/s41467-022-29366-6 PMC8976074 35365629

[54] Liu, R. et al. SEMA3C regulates tumor-associated macrophage phenotype and influences lung cancer cell migration and invasion through VNN1. *Hum. Exp. Toxicol*. **44**, 9603271251376577 (2025). 10.1177/09603271251376577 40901786

[55] Kubala, M. H. et al. Plasminogen activator inhibitor-1 promotes the recruitment and polarization of macrophages in cancer. *Cell Rep.* **25**, 2177 (2018). 10.1016/j.celrep.2018.10.082 PMC6876299 30463014

[56] Li, Y. et al. CAF-macrophage crosstalk in tumour microenvironments governs the response to immune checkpoint blockade in gastric cancer peritoneal metastases. *Gut* **74**, 350–363 (2025). 10.1136/gutjnl-2024-333617 PMC11874311 39537239

[57] Bueno, M. L. P., Saad, S. T. O. & Roversi, F. M. WNT5A in tumor development and progression: a comprehensive review. *Biomed. Pharmacother.* **155**, 113599 (2022). 10.1016/j.biopha.2022.113599 36089446

[58] Benabid, A. & Peduto, L. Mesenchymal perivascular cells in immunity and disease. *Curr. Opin. Immunol.* **64**, 50–55 (2020). 10.1016/j.coi.2020.03.009 PMC7597593 32387900

[59] He, B. et al. Selective tubulin-binding drugs induce pericyte phenotype switching and anti-cancer immunity. *EMBO Mol. Med.* **17**, 1071–1100 (2025). 10.1038/s44321-025-00222-6 PMC12081767 40140727

[60] Lei, X. et al. Targeting the NAT10/XIST/YAP1 axis-mediated vascular abnormalization enhances immune checkpoint blockade in gastric cancer. *Int. J. Biol. Sci.* **21**, 4997–5014 (2025). 10.7150/ijbs.113325 PMC12374829 40860183

[61] Li, Z.-J. et al. Pericyte phenotype switching alleviates immunosuppression and sensitizes vascularized tumors to immunotherapy in preclinical models. *J. Clin. Invest*. **134**, e179860 (2024). 10.1172/JCI179860 PMC11405053 39286984

[62] Longo, D. M. et al. Inter-donor variation in cell subset specific immune signaling responses in healthy individuals. *Am. J. Clin. Exp. Immunol.* **1**, 1 (2012). PMC3714187 23885311

[63] Wisniewski, T. et al. Assessment of chemokine receptor function on monocytes in whole blood: in vitro and ex vivo evaluations of a CCR2 antagonist. *J. Immunol. Methods* **352**, 101–110 (2010). 10.1016/j.jim.2009.10.010 19913021

[64] Weiss, J. M., Downie, S. A., Lyman, W. D. & Berman, J. W. Astrocyte-derived monocyte-chemoattractant protein-1 directs the transmigration of leukocytes across a model of the human blood–brain barrier. *J. Immunol.* **161**, 6896–6903 (1998). 9862722

[65] Valdor, R. et al. Glioblastoma ablates pericytes antitumor immune function through aberrant up-regulation of chaperone-mediated autophagy. *Proc. Natl Acad. Sci. USA* **116**, 20655–20665 (2019). 10.1073/pnas.1903542116 PMC6789971 31548426

[66] Braun, S. et al. Pericytes orchestrate a tumor-restraining microenvironment in glioblastoma. *Nat. Commun.* **16**, 10918 (2025). 10.1038/s41467-025-66985-1 PMC12680652 41339360

[67] Chen, Z. et al. Cellular and molecular identity of tumor-associated macrophages in glioblastoma. *Cancer Res.* **77**, 2266–2278 (2017). 10.1158/0008-5472.CAN-16-2310 PMC5741820 28235764

[68] Hosaka, K. et al. Pericyte-fibroblast transition promotes tumor growth and metastasis. *Proc. Natl Acad. Sci. USA* **113**, E5618–E5627 (2016). 10.1073/pnas.1608384113 PMC5035870 27608497

[69] Buechler, C. et al. Regulation of scavenger receptor CD163 expression in human monocytes and macrophages by pro- and antiinflammatory stimuli. *J. Leukoc. Biol.* **67**, 97–103 (2000). 10648003

[70] Mahon, O. R. et al. Extracellular matrix scaffolds derived from different musculoskeletal tissues drive distinct macrophage phenotypes and direct tissue-specific cellular differentiation. *J. Immunol. Regen. Med.* **12**, 100041 (2021).

[71] Chen, A. et al. Chitinase-3-like 1 protein complexes modulate macrophage-mediated immune suppression in glioblastoma. *J. Clin. Invest*. **131**, e147552 (2021). 10.1172/JCI147552 PMC8363281 34228644

[72] Guo, X. et al. Hypoxia promotes glioma-associated macrophage infiltration via periostin and subsequent M2 polarization by upregulating TGF-beta and M-CSFR. *Oncotarget* **7**, 80521–80542 (2016). 10.18632/oncotarget.11825 PMC5348338 27602954

[73] Chomarat, P., Banchereau, J., Davoust, J. & Palucka, A. K. IL-6 switches the differentiation of monocytes from dendritic cells to macrophages. *Nat. Immunol.* **1**, 510–514 (2000). 10.1038/82763 11101873

[74] Zhang, X. N. et al. Pericytes augment glioblastoma cell resistance to temozolomide through CCL5–CCR5 paracrine signaling. *Cell Res.* **31**, 1072–1087 (2021). 10.1038/s41422-021-00528-3 PMC8486800 34239070

[75] Oudenaarden, C., Sjölund, J. & Pietras, K. Upregulated functional gene expression programmes in tumour pericytes mark progression in patients with low-grade glioma. *Mol. Oncol.* **16**, 405–421 (2022). 10.1002/1878-0261.13016 PMC8763650 34018679

