# Supplementary material for: FAP+ pericyte-like cells promote monocyte differentiation into tumor-associated macrophages in glioblastoma
Source: Exp Mol Med. 2026 Sep 11;58(9):3177–95. doi: 10.1038/s12276-026-01827-8 (PMC13633274; doi:10.1038/s12276-026-01827-8)
Supplement: Supplementary file 1 [file 12276_2026_1827_MOESM1_ESM.pdf]

FAP+ pericyte-like cells promote monocyte differentiation into tumor-associated macrophages in glioblastoma

Magdalena Houdova Megova[1], Chloe Shard[2,+], Barbora Vymolova[1,+], Nikola Ternerova[1], Tereza Svablova[1], Tomas Buna [1], Daniel Straka [1], Zdislava Vanickova[1], Evzen Krepela[1*], Helena Kupcova Skalnikova [1], Michal Kolar[3], Jana Sachova[3], Jan Kubovciak[3], Eva Balaziova[1], Petr Vymola[1], Petr Hrabal[4], Lisa M. Ebert[2,5,6], Ashwini Patil[7], Robert Tomas[8], David Netuka[9], Guillermo A. Gomez[2], Petr Busek[1], Aleksi Sedo[1]

[1] Laboratory of Cancer Cell Biology, Institute of Biochemistry and Experimental Oncology, First Faculty of Medicine, Charles University, 128 00 Prague, Czech Republic

[2] Centre for Cancer Biology, Adelaide University, Adelaide, SA 5000, Australia.

[3] Laboratory of Genomics and Bioinformatics, Institute of Molecular Genetics of the Czech Academy of Sciences, 142 20 Prague, Czech Republic.

[4] Department of Pathology, Military University Hospital, 169 02 Prague, Czech Republic

[5] Adelaide Medical School, University of Adelaide, Adelaide, SA 5000, Australia.

[6] Cancer Clinical Trials Unit, Royal Adelaide Hospital, Adelaide, SA 5000, Australia.

[7] Combinatics, Chiba, 272-0824, Japan.

[8] Department of Neurosurgery, Motol and Homolka University Hospital, 150 00 Prague, Czech Republic

[9] Department of Neurosurgery and Neurooncology, First Faculty of Medicine, Charles University and Military University Hospital, 168 02 Prague, Czech Republic

* Current address: Institute of Medical Biochemistry and Laboratory Diagnostics of the General University Hospital and the First Faculty of Medicine, Charles University, Prague, Czech Republic
+ contributed equally

*** Correspondence:**
Petr Busek
Laboratory of Cancer Cell Biology, Institute of Biochemistry and Experimental Oncology, First Faculty of Medicine, Charles University, U Nemocnice 5, 128 00 Prague, Czech Republic
+420224965825
petr.busek2@lf1.cuni.cz

Guillermo A. GomezCentre for Cancer Biology, Adelaide University, Adelaide, SA 5000, Australia.
guillermo.gomez@adelaide.edu.au

Supplementary table and figure legends

Human transcript	GenBank Acession Number		Sequences of primers and TaqMan probes
**Primers and TaqMan probes used for the relative quantification of immune cell-related transcripts using real-time RT-PCR.**			
β-Actin (ACTB)	NM_001101.2	Forward primer	5'-ATGGCCACGGCTGCTT-3'
		Reverse primer	5'-CCATGCCCAGGAAGGAA-3'
		TaqMan probe	5'-(6-FAM)CCCTGGAGAAGAGCTACGAGCTGCCT(TAMRA)-3'
Glyceraldehyde-3-phosphate dehydrogenase (GAPDH)	NM_002046.4	Forward primer	5'-GGGTGTGAACCATGAGAAGTATG-3'
		Reverse primer	5'-TGGACTGTGGTCATGAGTCCT-3
		TaqMan probe	5'-(6-FAM)CATCAGCAATGCCTCCTGCACCAC(TAMRA)-3'
β-Tubulin (TUBB)	NM_178014.2	Forward primer	5'-CTGGACCGCATCTCTGTGTAC-3'
		Reverse primer	5'-CCAGACTGACCAAATACAAAGTTG-3'
		TaqMan probe	5'-(6-FAM)TGGCACGAGGAACATATTTGCCACCT(TAMRA)-3'
CD40	NM_001250.5	Forward primer	5'-CCTGCACCGCTCATGCT-3'
		Reverse primer	5'-TTGGTCTCACAGCTTGTCCAA-3'
		TaqMan probe	5'-(6-FAM)CTGCCCAGTCGGCTTCTTCTCCAA(TAMRA)-3'
CD68	NM_001251.3	Forward primer	5'-CAGACAGCCTAGCTGGACTTTG-3'
		Reverse primer	5'-CCCTGGGCTGCCAGTA-3'
		TaqMan probe	5'-(6-FAM)CGAGAAAAGCACAGCCAGCCTCATG(TAMRA)-3'
CD74	NM_001025159.2	Forward primer	5'-CGCTCCACCGAAAGTACTGA-3'
		Reverse primer	5'-CCAGTGACTCACTGCAGTTATGG-3'
		TaqMan probe	5'-(6-FAM)CATTCAGGCCCAAGTGCGACGAG(TAMRA)-3'
CD163	NM_004244.5	Forward primer	5'-AAGAGGAGAGAACTTAGTCCACCAA-3'
		Reverse primer	5'-ACTCATGGGAATTTTCTGAGGAA-3'
		TaqMan probe	5'-(6-FAM)TCAGGCAAGAATTCATCTCCCG(TAMRA)-3'
Macrophage scavenger receptor-1 (CD204)	NM_138715.2	Forward primer	5'-GTGACGATCGCTGGGAAGT-3'
		Reverse primer	5'-GACCAGTACCTTGTCCAAAGTGAG-3'
		TaqMan probe	5'-(6-FAM)ACCTGGGTATCCCAAGCTCCTACAGACG(TAMRA)-3'
Fibroblast activation protein (FAP)	NM_004460.4	Forward primer	5'-TCTGCTGTGCTTGCCTTATTG-3'
		Reverse primer	5'-ATGAAGATATTCTTGTCCTGAAATCC-3'
		TaqMan probe	5'-(6-FAM)TGCATTGTCTTACGCCCTTCAAGAGTTCA(TAMRA)-3'

Supplementary Table 1. List of primers and TaqMan probes used for the relative quantification of immune cell-related transcripts using real-time RT-PCR.

Transport_Pericytes	ECM_Pericytes	Interferon_Pericytes	SMCs	FAP_Pericytes	Proliferative_Pericytes
ITIH5	PRR16	CSF2RA	MYH11	POSTN	ASPM
P2RY14	PIEZO2	SAMSN1	CSDC2	CA12	PBK
SLC6A13	PRKG1	CD53	DES	CXCL6	AURKB
SLC6A12	THY1	DOCK2	AC097724.3	FBLN2	UBE2C
COL4A3	FAT1	ADAM28	PLN	CYP1B1	FAM64A
ADARB2	CD36	PTPRC	LMOD1	FAP	KIF2C
SLC30A10	IL1RAPL1	SLA	CNN1	COL11A1	SKA1
SLC19A1	NREP	RBM47	NET1	PDGFRA	SHCBP1
DIO3OS	C2orf27A	HLA-DRB1	MYOCD	SFRP2	CENPF
PTGDR2	BMP1	VSIG4		FLRT2	RRM2
SHISA6	TGFBI	RNF144B		THBS2	KIF23
	TUSC3	ITGB2		SGCD	CDC20
	DOK6	PLEK		FMOD	KIF11
	EBF2	SLC11A1		LTBP2	TOP2A
		FYB		CLDN11	KIF15
		HLA-DQB1		GAS7	MAD2L1
		CD86		PDPN	DLGAP5
		MSR1		ELN	CENPM
		ITGAX		OGN	GTSE1
		SLC16A10		CLMP	CDCA8
		MS4A4A		BICC1	ANLN
		SYK		ROR2	TPX2
		NLRP3		SFRP4	KIF14
		FCGR3A		GJA1	CCNA2
		AOAH		OMD	NUF2
		FCER1G			KIF18B
		C1QC			CEP55
		LCP1			CCNB1
					CKAP2L
					DIAPH3
					TK1
					SGOL1
					NUSAP1

Supplementary Table 2. Pericyte subpopulation markers used to annotate scRNA-seq pericyte clusters.

Association between the FAP⁺ pericyte transcriptional signature and overall survival in IDH-wildtype primary glioblastoma.

Cohort	Variable	Comparison	Model	Covariate	N	Events	HR	95% CI	p-value
TCGA RNA-seq	Signature score	per 1 unit increase	Unadjusted	-	145	118	1.282	0.968–1.697	0.083
	Signature score	per 1 unit increase	Age-adjusted	Age	145	118	1.238	0.933–1.643	0.139
	Signature terciles	Upper vs Lower	Unadjusted	-	97	79	1.608	1.013–2.552	0.044
	Signature terciles	Upper vs Lower	Age-adjusted	Age	97	79	1.531	0.963–2.433	0.072
TCGA HG-U133A	Signature score	per 1 unit increase	Unadjusted	-	482	381	1.160	1.001–1.344	0.049
	Signature score	per 1 unit increase	Age-adjusted	Age	482	381	1.139	0.982–1.322	0.086
	Signature terciles	Upper vs Lower	Unadjusted	-	322	258	1.152	0.902–1.473	0.257
	Signature terciles	Upper vs Lower	Age-adjusted	Age	322	258	1.079	0.844–1.379	0.544
CGGA mRNAseq_325	Signature score	per 1 unit increase	Unadjusted	-	74	64	1.417	1.026–1.956	0.034
	Signature score	per 1 unit increase	Age-adjusted	Age	74	64	1.358	0.984–1.873	0.063
	Signature terciles	Upper vs Lower	Unadjusted	-	50	42	2.547	1.306–4.969	0.006
	Signature terciles	Upper vs Lower	Age-adjusted	Age	50	42	2.838	1.396–5.769	0.004
CGGA mRNAseq_693	Signature score	per 1 unit increase	Unadjusted	-	105	92	0.753	0.549–1.033	0.079
	Signature score	per 1 unit increase	Age-adjusted	Age	105	92	0.721	0.523–0.995	0.047
	Signature terciles	Upper vs Lower	Unadjusted	-	70	60	0.598	0.355–1.007	0.053
	Signature terciles	Upper vs Lower	Age-adjusted	Age	70	60	0.548	0.323–0.931	0.026

Hazard ratio (HR) estimates from Cox proportional hazards regression analyses evaluating the association between the FAP⁺ pericyte signature score and overall survival across independent cohorts (TCGA RNA-seq, TCGA HG-U133A, CGGA mRNAseq_325, and CGGA mRNAseq_693). The signature score was modeled as a continuous variable (per unit increase in the mean of gene-wise z-scores) and as a categorical variable (upper vs lower tercile). Unadjusted and age-adjusted models are presented. N indicates the number of patients included in each model; Events indicate the number of deaths. 95% CI denotes the 95% confidence interval of the hazard ratio. HR values >1 indicate increased risk associated with higher signature score.

Supplementary Table 4. Association between the FAP⁺ pericyte transcriptional signature and overall survival in IDH-wildtype primary glioblastoma.

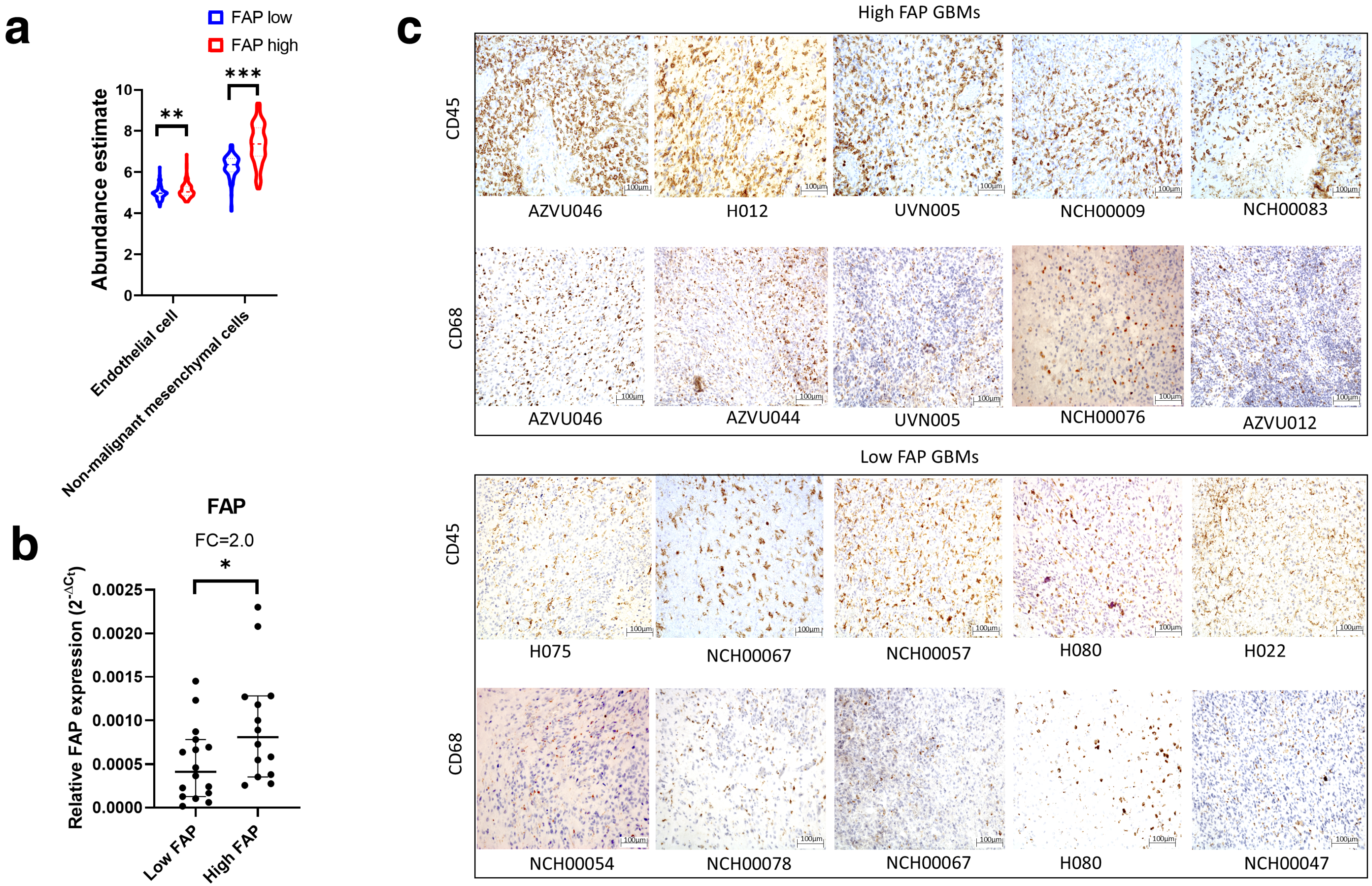

Supplementary Fig. 1. a Proportion of endothelial cells and non-malignant mesenchymal cells in FAP high and FAP low GBMs in the TCGA GBM dataset determined using MCP Counter. $^{**}p < 0.01$, $^{***}p < 0.001$, Mann-Whitney U test. **b** Relative expression of FAP transcript in FAP high and FAP low GBMs. $^{*}p < 0.05$, Mann-Whitney U test. Line = median, whiskers = 95 % confidence interval, FC = fold change. **c** Representative images of CD45 and CD68 immunohistochemistry staining in five low FAP and five high FAP GBM tissues.

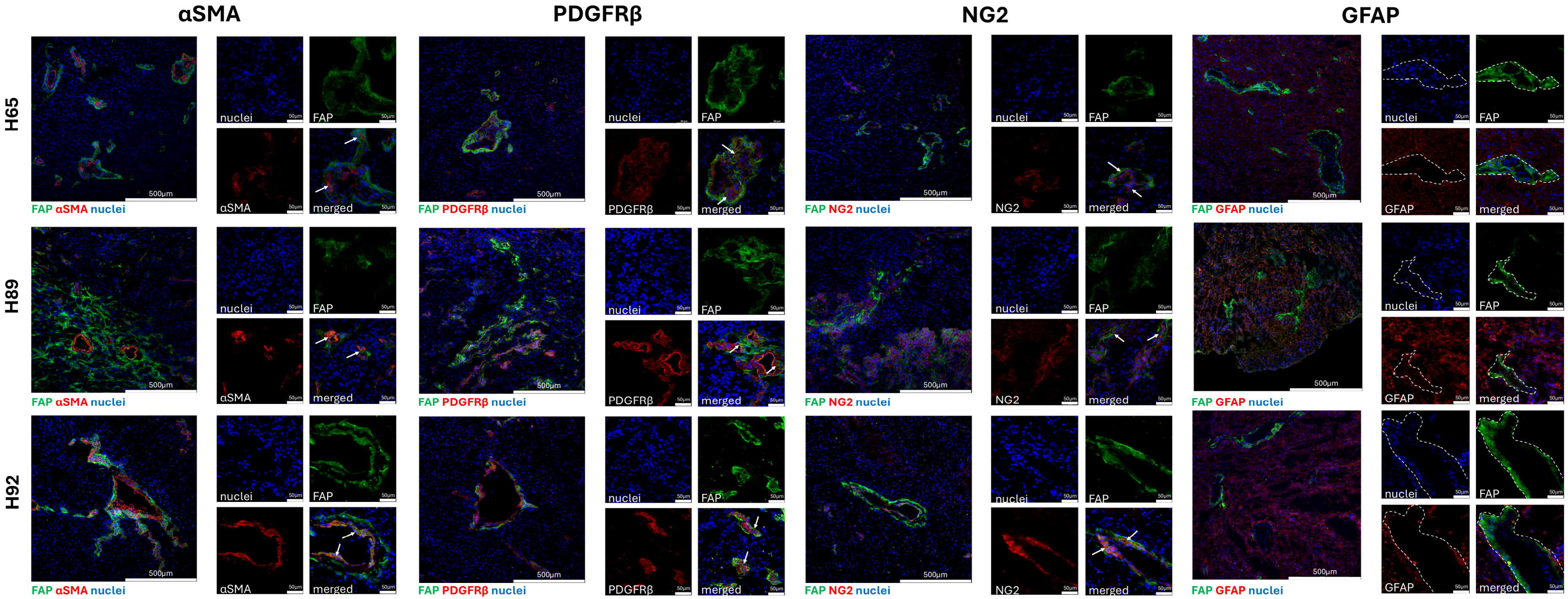

Supplementary Fig. 2. Perivascular localization of FAP⁺ cells in human glioblastoma.

Representative immunofluorescence images from three glioblastomas with high FAP expression are shown. FAP$^+$ cells are predominantly localized to perivascular regions and exhibit overlapping immunoreactivity with mesenchymal/perivascular markers α–smooth muscle actin (αSMA), PDGFRβ, and NG2. Arrows indicate double-positive cells. In contrast, FAP expressing cells in perivascular regions do not exhibit overlap with the glial marker GFAP (glial fibrillary acidic protein). Dotted lines delineate regions of GFAP and FAP positivity.

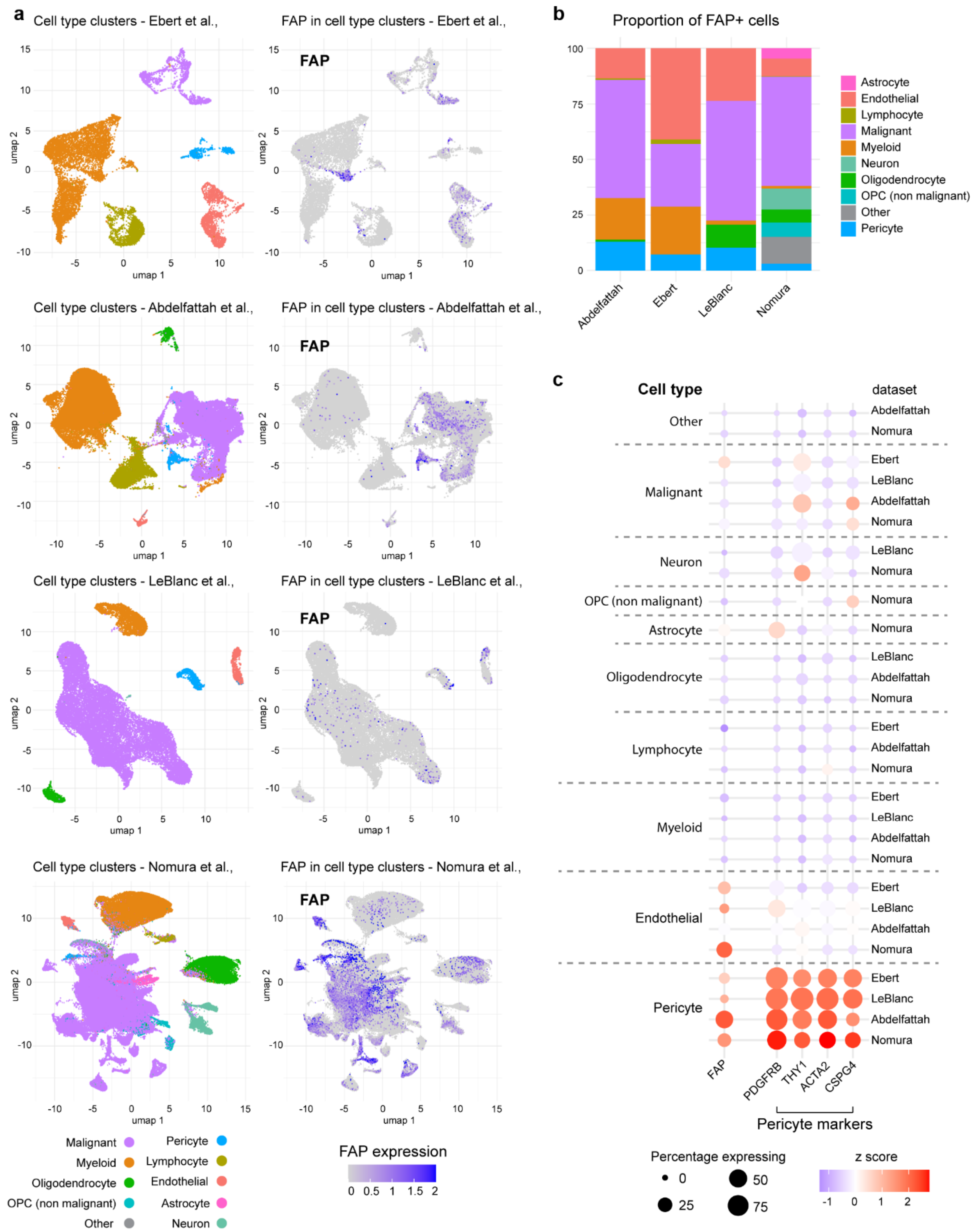

Supplementary Fig. 3. a UMAP plots depicting FAP expression across individual cell types in the Ebert, Abdelfattah, LeBlanc, and Nomura scRNA-seq datasets[1–4]. **b** Proportion of annotated cell types among FAP$^+$ cell in each dataset (per patient normalized), illustrating that FAP transcripts are detectable in multiple cell populations in addition to pericytes, consistent with previous reports[1]. Differential expression of myeloid cell-modulating ligands across FAP$^+$ cell populations is presented in Supplementary Fig. 5a. **c** Expression of FAP and canonical pericyte markers (PDGFRB, THY1, ACTA2, and CSPG4) across annotated cell populations in the individual scRNA-seq datasets confirms correct

annotation of pericytes in individual dataset. Dot size indicates the percentage of expressing cells, and color intensity represents scaled expression (z score). Cell type annotations were adopted from the original publications.

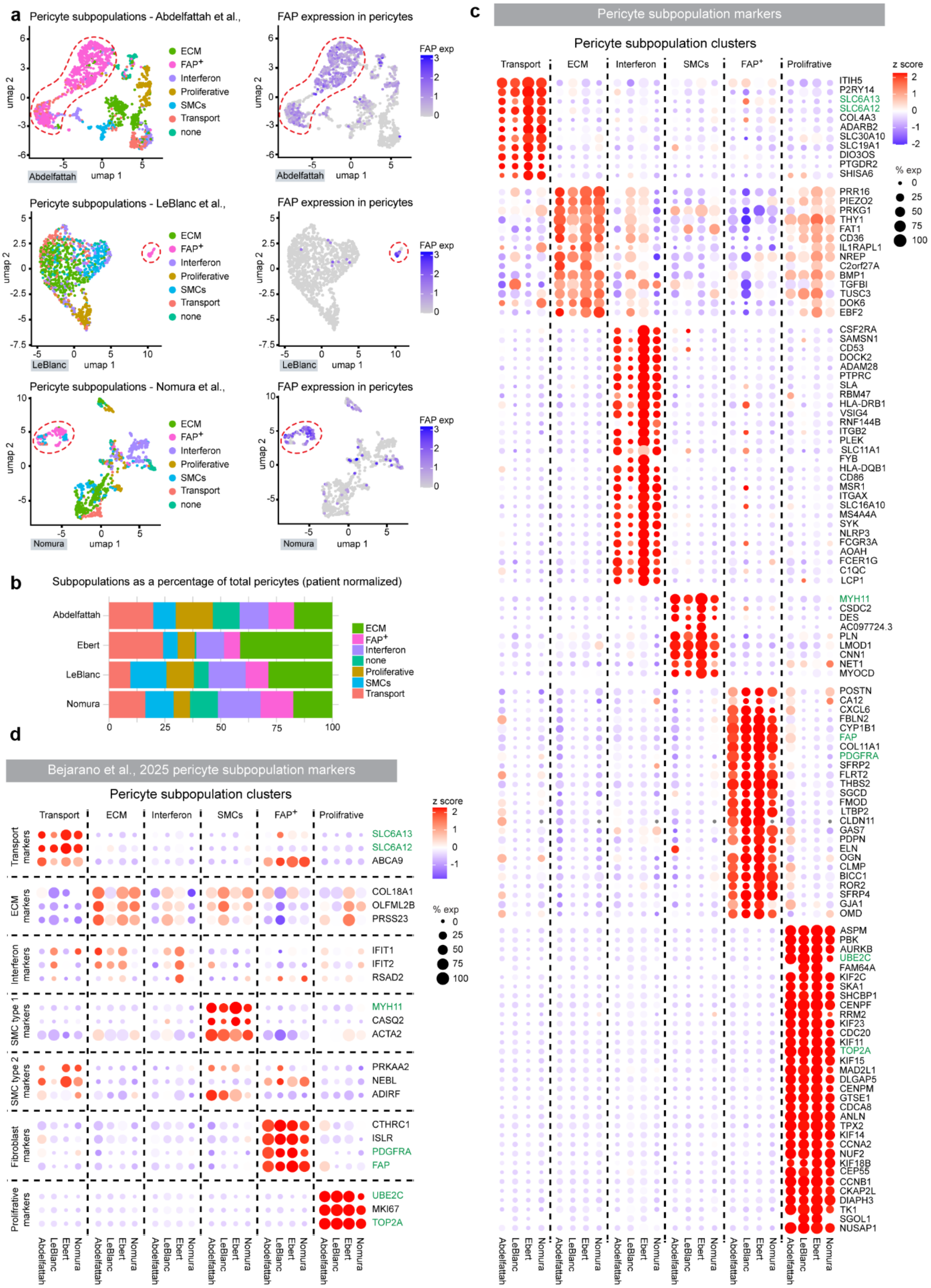

Supplementary Fig. 4. a UMAPs of the pericyte subpopulations and FAP expression in the Abdelfattah, Leblanc, and Nomura datasets. **b** Proportion of each pericyte subpopulation as a percentage of total pericytes in each dataset (per patient normalized). **c** Dot plot of pericyte subpopulation marker expression across pericyte subpopulations annotated in four primary glioblastoma (GBM) scRNAseq datasets [1–4]comprising a total of 79 GBM tumor biopsies. **d** Dot plot of pericyte subpopulation markers defined by Bejarano et al., [5] in pericyte subpopulations annotated by markers defined in panel C in the Ebert, Abdelfattah, LeBlanc, and Nomura datasets. Dot size indicates the percentage of expressing cells, and color intensity represents scaled expression (z score).

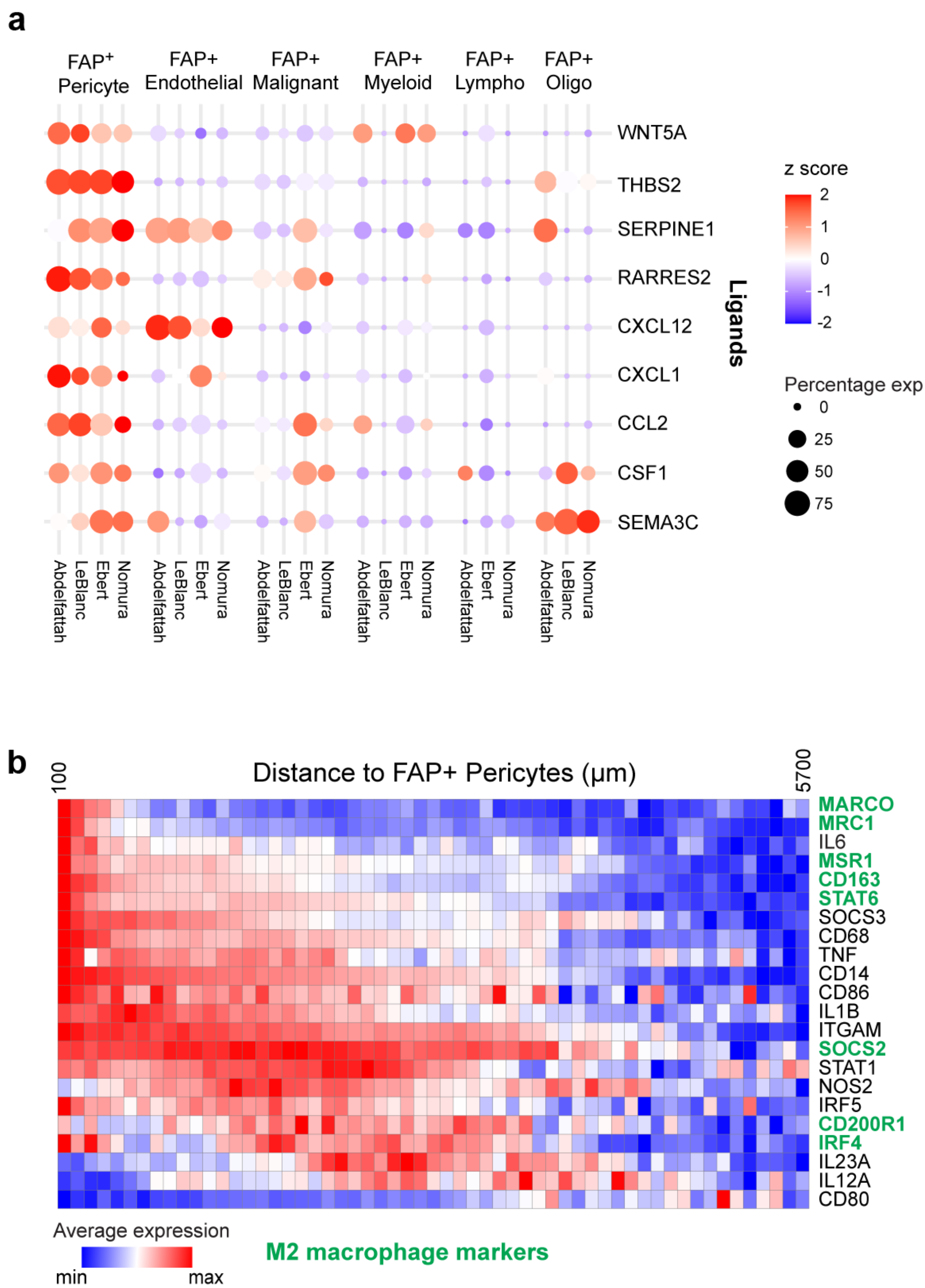

Supplementary Fig. 5. a Dot plots showing the expression of key immune-regulatory ligands across cell populations in individual scRNA-seq datasets. Many ligands show preferential expression in FAP⁺ pericytes, even when compared with other FAP⁺ cell populations in GBM including malignant, endothelial, and myeloid cells, lymphocytes, and oligodendrocytes. Only FAP⁺ subpopulations of each

cell type are shown. Dot size indicates the percentage of expressing cells, and color intensity represents scaled expression (z score). Cell type annotations were adopted from the original publications. While endothelial, myeloid and malignant cells were consistently present in all four datasets, lymphocytes and oligodendrocytes were not detected across all datasets based on original author annotations (Supplementary Fig. 3a). FAP$^+$ oligodendrocytes and FAP$^+$ lymphocytes were scarce in the scRNA-seq datasets (Supplementary Fig. 3b). **b** Heatmap illustrating the average expression level of myeloid marker genes as a function of distance (µm) from FAP$^+$ pericyte-enriched spots. M2 macrophage markers MARCO, MRC1, MSR1, CD163, and STAT6 are enriched proximal to FAP$^+$ pericytes. M2 macrophage markers are highlighted in green. Spatial transcriptomics analyses were performed on 13 vessel-enriched tissue sections from 10 GBM patients (see methods) obtained from two public datasets. [6,7]

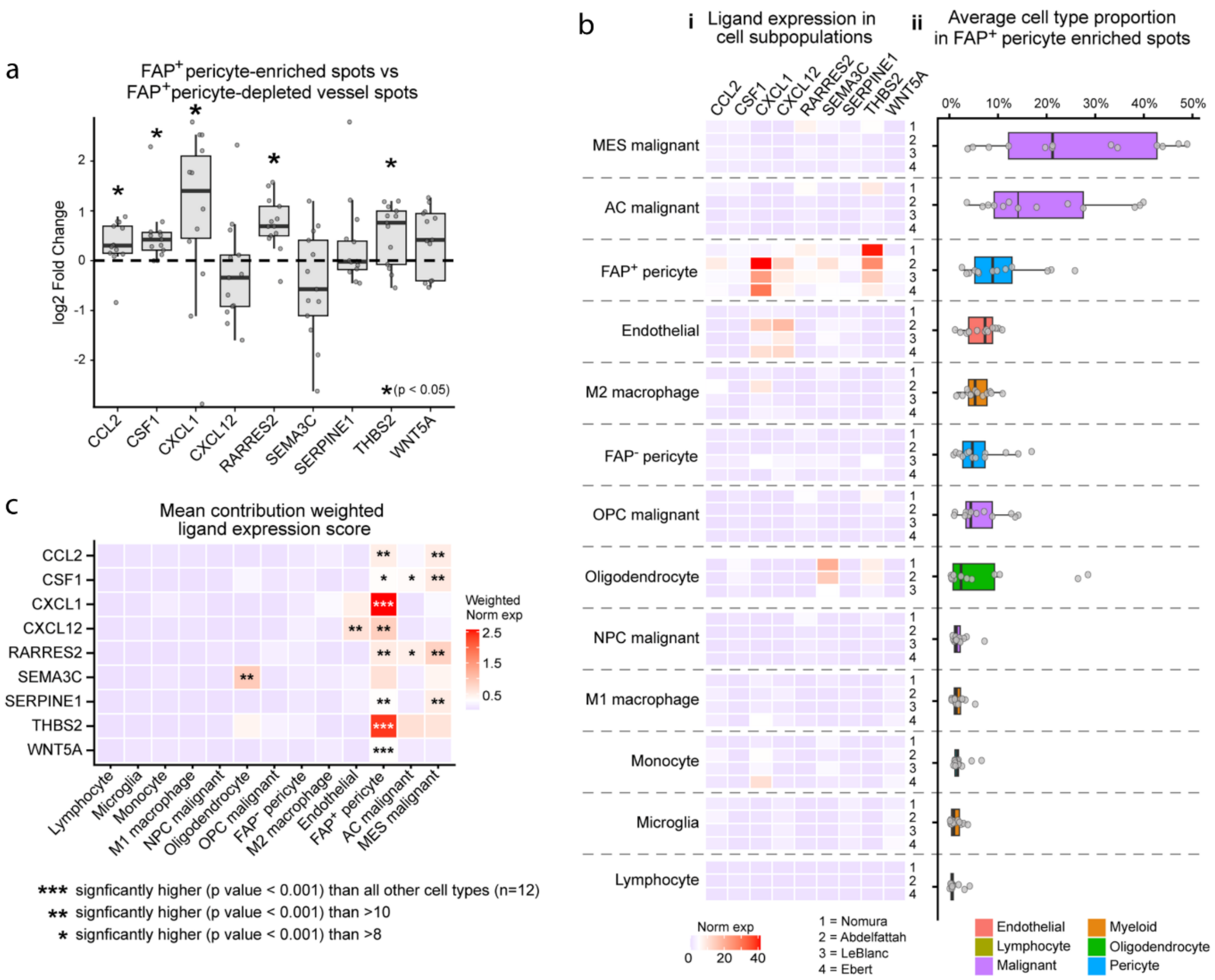

Supplementary Fig. 6. Ligand enrichment in FAP$^+$ pericyte niches. **a** Spatial transcriptomics analysis of ligand expression in FAP$^+$ pericyte-enriched spots compared to FAP$^+$ pericyte-depleted vessel spots. Log_2 fold-change values were calculated for individual tissues. Line – median; box – interquartile range (25th - 75th percentile); whiskers – non-outlier values; dots – individual tissues, * $p < 0.05$ paired t-test. **b** Dataset-specific normalized ligand expression score and cell-type composition in FAP$^+$ pericyte enriched spots. (i) Heatmap showing ligand expression scores calculated within each annotated cell subtype and normalized to the global mean expression of the corresponding ligand across all cells within each scRNA-seq dataset. (ii) Average cell-type proportions in FAP$^+$ pericyte-enriched spots determined by robust cell type decomposition (RCTD). Numbers indicate the individual scRNA-seq datasets (1, Nomura; 2, Abdelfattah; 3, LeBlanc; 4, Ebert). MES, AC, NPC, OPC malignant cell subpopulations were defined using the gene lists provided in Neftel et al., (2019), see methods. **c** Contribution-weighted ligand expression score in FAP$^+$ pericyte-enriched spots. Average normalized

ligand expression for each cell subtype was calculated across the four scRNA-seq datasets and multiplied by the mean cell-type proportion in FAP⁺ pericyte-enriched spots. Heatmap values represent the resulting contribution-weighted expression scores. Statistical significance was assessed by one-way ANOVA with Tukey-adjusted multiple-comparison testing. Asterisks denote ligand–cell subtype combinations with significantly higher contribution-weighted expression scores than other cell subtypes ($p < 0.001$).

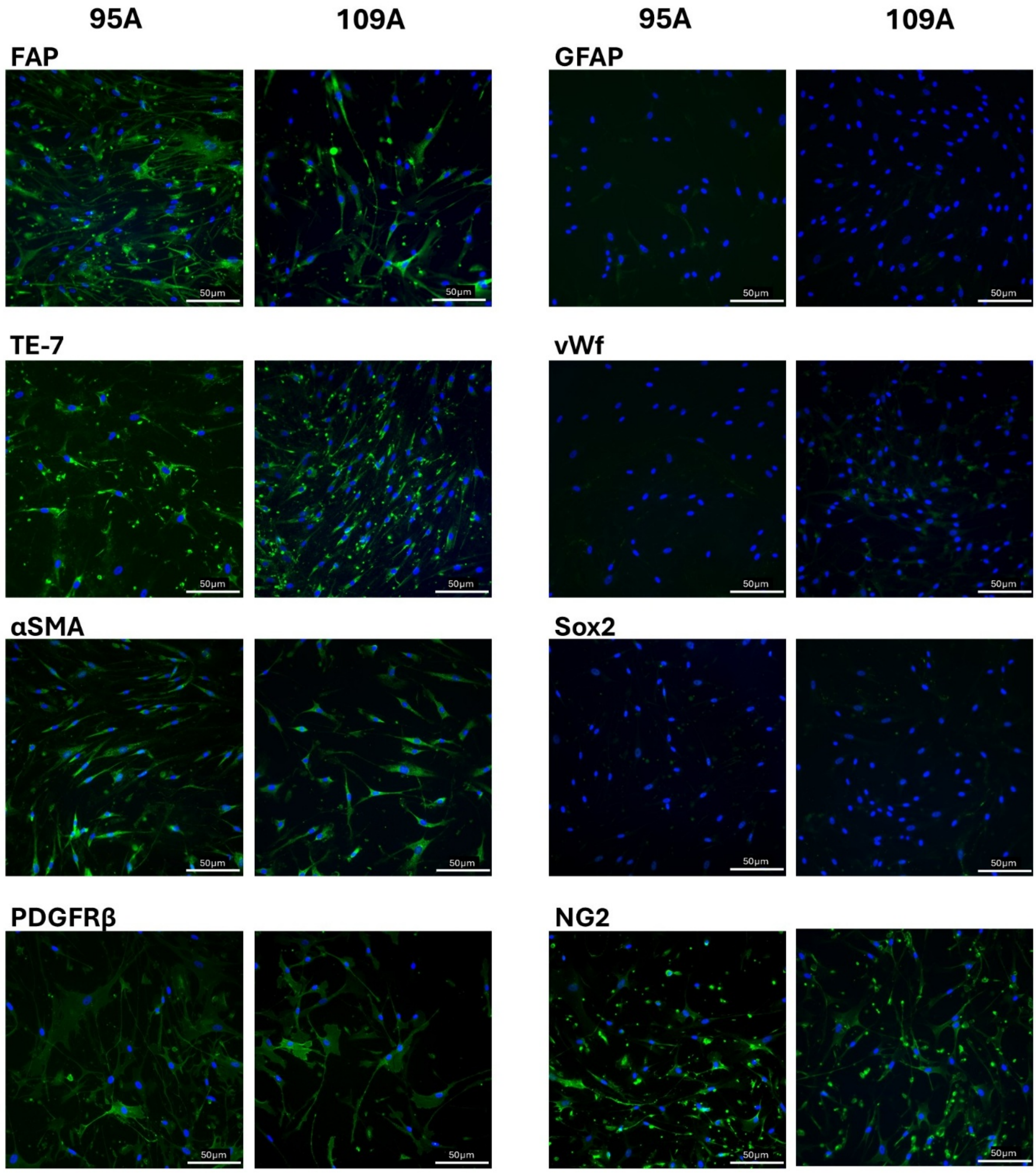

Supplementary Fig. 7. Characterization of primary FAP⁺ pericyte-like cell cultures by immunocytochemistry. Primary FAP⁺ pericyte-like cell cultures derived from human glioblastomas express mesenchymal markers and lack markers associated with malignant glial and endothelial cell populations. Representative immunocytochemistry (ICC) images of cultures 95A and 109A demonstrate expression of mesenchymal markers (FAP, TE-7, αSMA, PDGFRβ) and absence of endothelial (CD31, vWf) and glial (GFAP) markers.

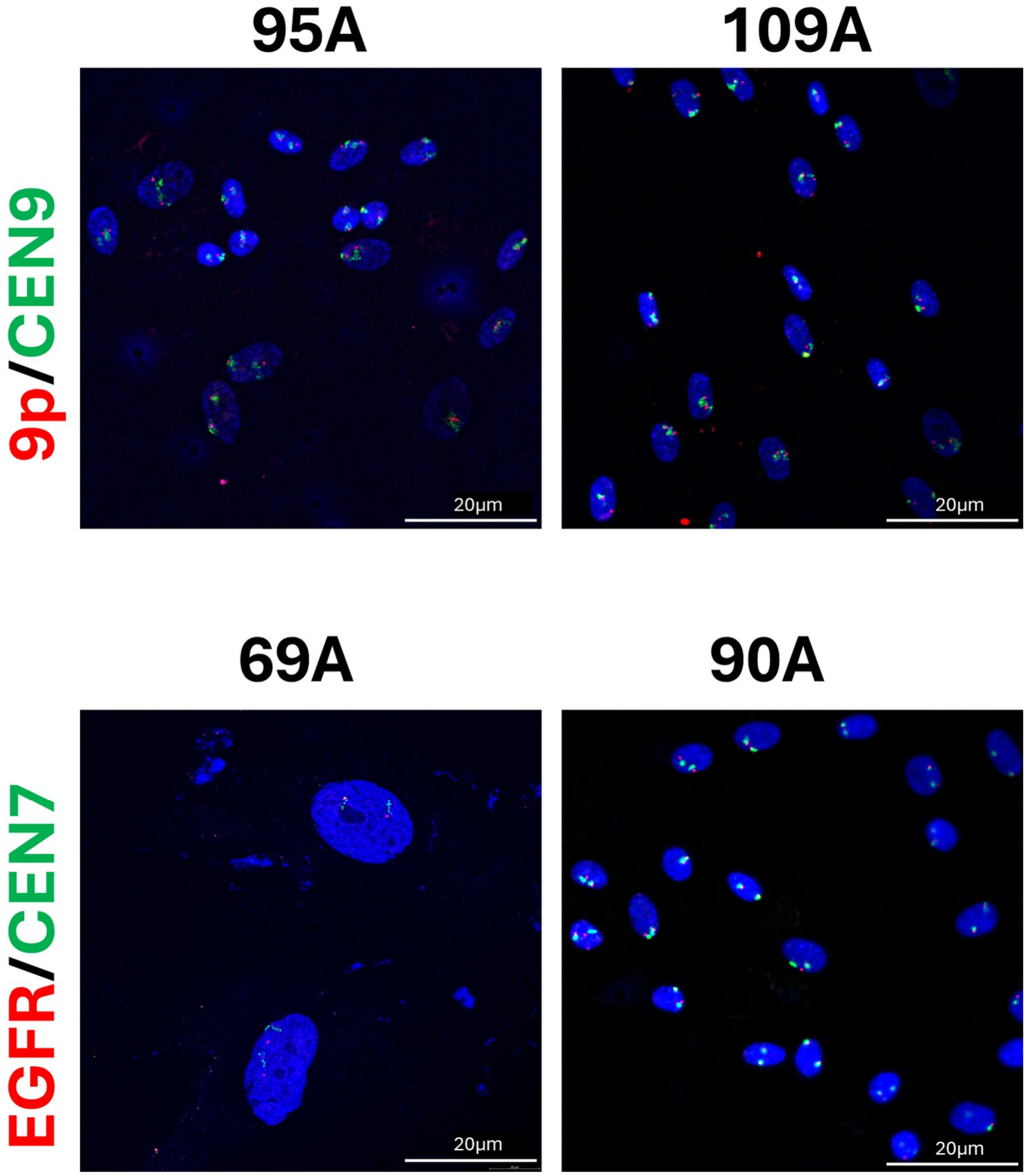

Supplementary Fig. 8. Fluorescence *in situ* hybridization in primary FAP⁺ pericyte-like cell cultures. Primary FAP⁺ pericyte-like cell cultures derived from human glioblastomas lack the genetic alterations detected in the corresponding patient tumor tissues. Representative fluorescence *in situ* hybridization (FISH) images of cultures 95A, 109A, 69A, and 90A are shown. Dual-color probes targeting the CDKN2A (9p21) locus (orange) with centromere 9 (CEN9, green) control, or the EGFR locus (orange) with centromere 7 (CEN7, green) control, were used. Nuclei were counterstained with DAPI or Hoechst.

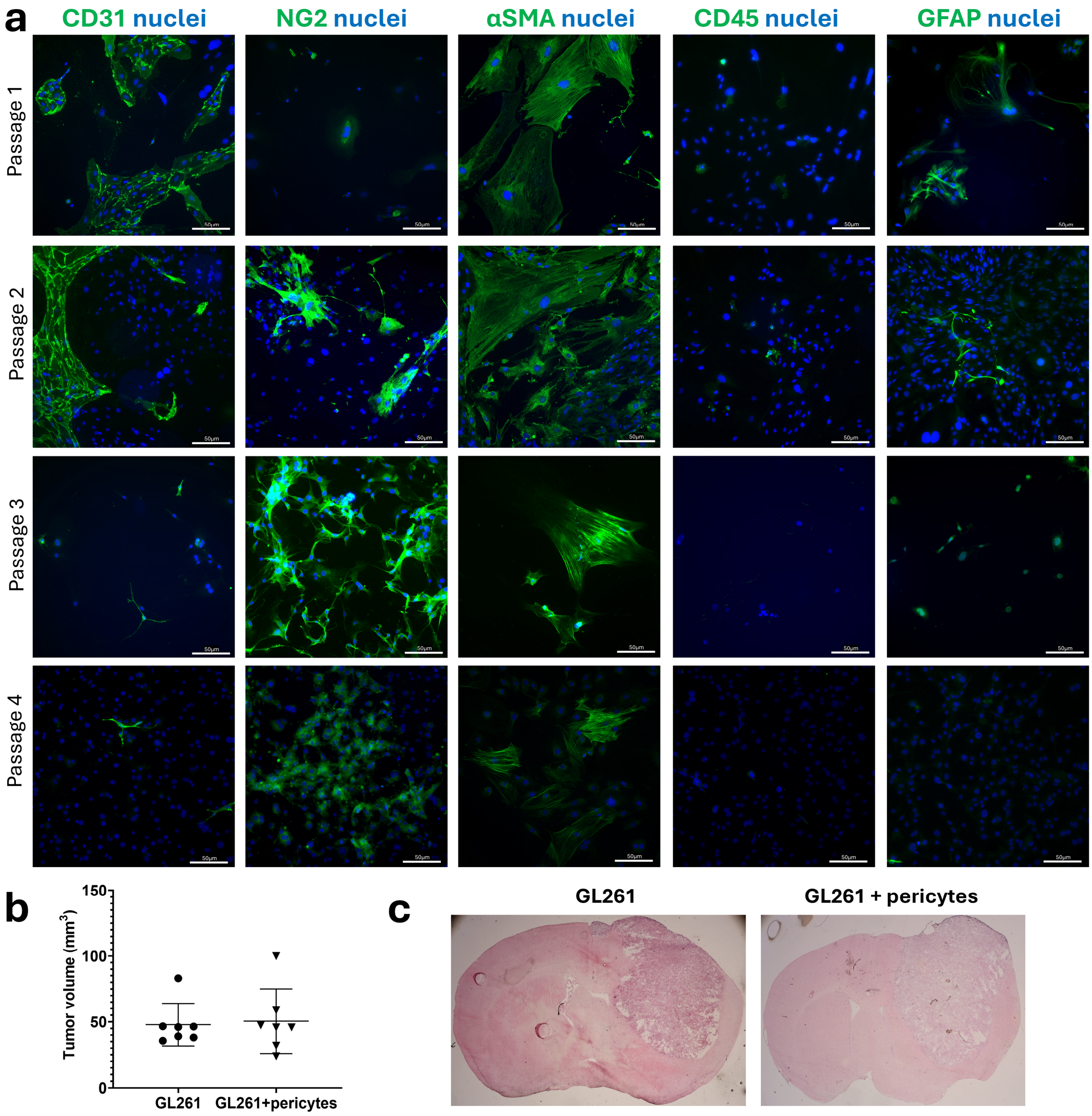

Supplementary Fig. 9. Phenotypic characterization of primary mouse brain–derived pericytes across passages and tumor volumetric analysis in the GL261 co-implantation model. **a** Immunocytochemical characterization of primary mouse brain–derived pericytes across serial passages. Representative images show cultures at passages 1–4 (rows) stained for endothelial (CD31), pericyte-associated (NG2, αSMA), hematopoietic (CD45), and astrocytic (GFAP) markers (columns). From passage 3 onward, cultures display a homogeneous pericytic phenotype. Nuclei were counterstained with Hoechst. Scale bars, 50 µm. **b** Tumor volume measurements of GL261 glioblastomas implanted alone or co-implanted with primary mouse brain–derived pericytes. Tumor volumes were calculated from serial hematoxylin and eosin (H&E)–stained coronal sections. **c** Representative H&E-stained coronal brain sections from mice bearing GL261 tumors alone or GL261 tumors co-implanted with primary mouse brain–derived pericytes.

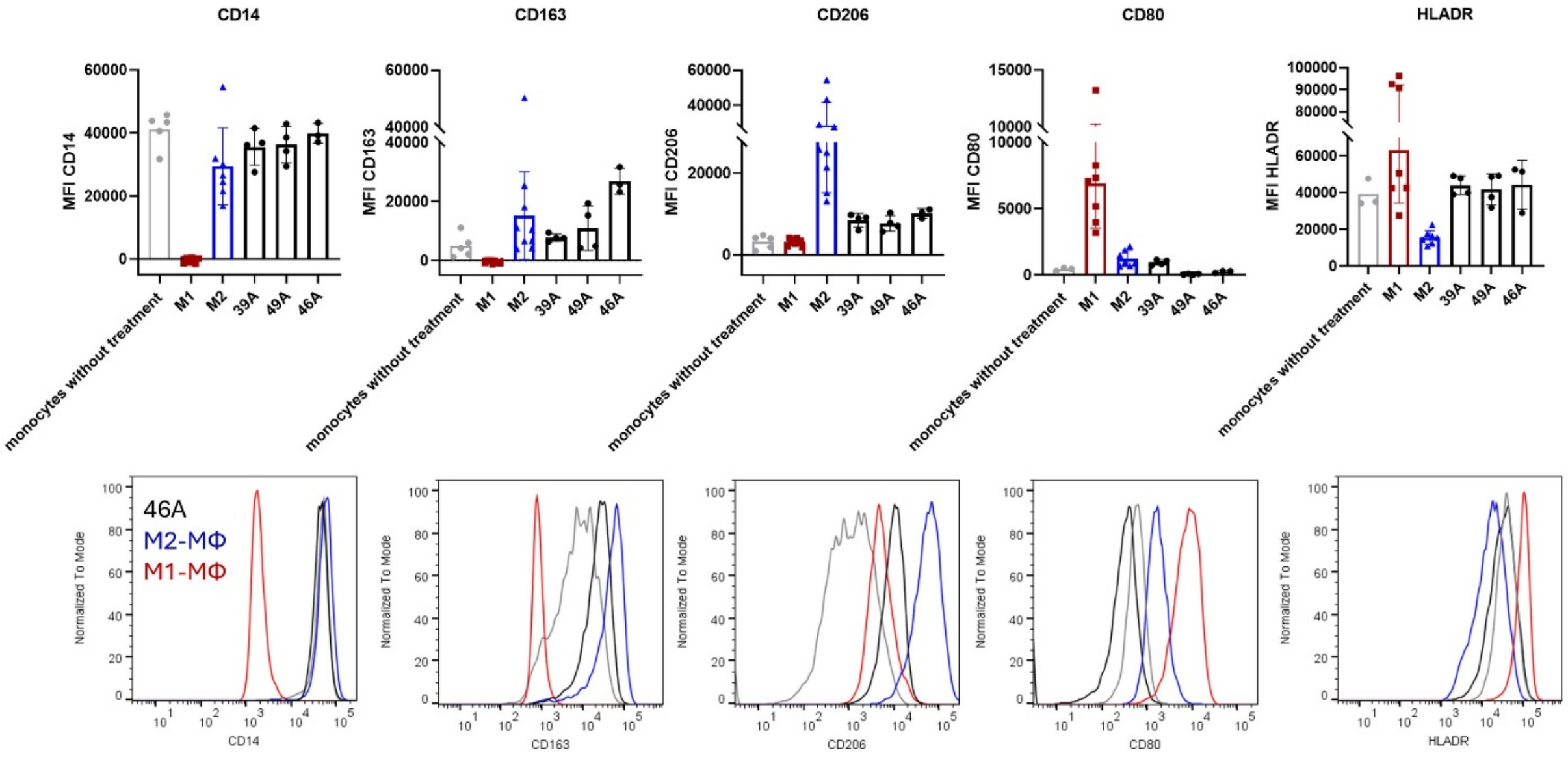

Supplementary Fig. 10. Expression of macrophage polarization markers determined by flow cytometry and representative histograms of monocytes co-cultured with FAP⁺ mesenchymal cultures (39A, 49A, 46A), classically (M1) and alternatively (M2) polarized macrophages, and monocytes without any treatment. MFI = mean fluorescence intensity.

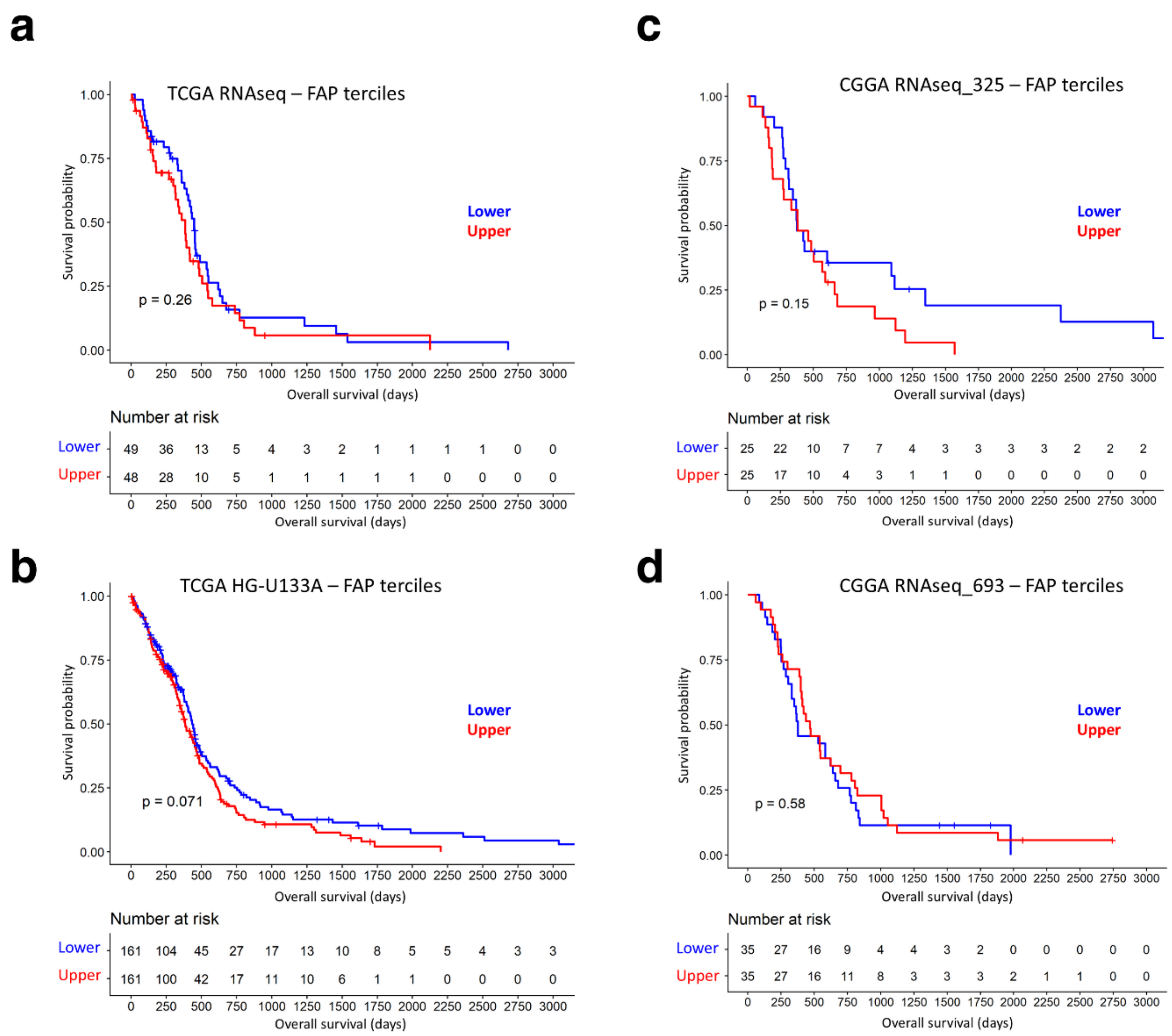

e

Cohort	Variable	Comparison	Model	Covariate	N	Events	HR	95% CI	p-value
TCGA RNA-seq	FAP (z-score)	per 1 SD increase	Unadjusted	-	145	118	1.159	0.954–1.408	0.136
	FAP (z-score)	per 1 SD increase	Age-adjusted	Age	145	118	1.077	0.879–1.320	0.472
	FAP terciles	Upper vs Lower	Unadjusted	-	97	79	1.291	0.826–2.019	0.262
	FAP terciles	Upper vs Lower	Age-adjusted	Age	97	79	1.091	0.683–1.745	0.715
TCGA HG-U133A	FAP (z-score)	per 1 SD increase	Unadjusted	-	482	381	1.092	0.991–1.203	0.075
	FAP (z-score)	per 1 SD increase	Age-adjusted	Age	482	381	1.069	0.971–1.178	0.173
	FAP terciles	Upper vs Lower	Unadjusted	-	322	250	1.261	0.980–1.622	0.072
	FAP terciles	Upper vs Lower	Age-adjusted	Age	322	250	1.191	0.925–1.533	0.176
CGGA RNAseq_325	FAP (z-score)	per 1 SD increase	Unadjusted	-	74	64	1.217	0.955–1.552	0.112
	FAP (z-score)	per 1 SD increase	Age-adjusted	Age	74	64	1.160	0.905–1.487	0.241
	FAP terciles	Upper vs Lower	Unadjusted	-	50	45	1.564	0.850–2.877	0.151
	FAP terciles	Upper vs Lower	Age-adjusted	Age	50	45	1.756	0.926–3.331	0.085
CGGA RNAseq_693	FAP (z-score)	per 1 SD increase	Unadjusted	-	105	92	0.907	0.747–1.102	0.327
	FAP (z-score)	per 1 SD increase	Age-adjusted	Age	105	92	0.865	0.711–1.053	0.150
	FAP terciles	Upper vs Lower	Unadjusted	-	70	65	0.870	0.534–1.420	0.578
	FAP terciles	Upper vs Lower	Age-adjusted	Age	70	65	0.807	0.490–1.328	0.399

Supplementary Fig. 11. FAP expression and overall survival in IDH-wildtype primary glioblastoma. Kaplan–Meier survival curves for patients stratified by terciles of FAP expression in the **a** TCGA RNA-seq, **b** expanded TCGA HG-U133A, **c** CGGA RNA-seq_325, and **d** CGGA RNA-seq_693 cohorts. Within each cohort, FAP expression was z-score normalized prior to tercile stratification. P values shown in panels **a–d** are derived from two-sided log-rank tests. **e** Summary table of hazard ratio (HR) estimates from Cox proportional hazards regression analyses evaluating the association between FAP expression and overall survival across IDH-wildtype primary glioblastoma cohorts. FAP expression was modeled as

a continuous variable (per 1 standard deviation increase in z–scored expression) and as a categorical variable (upper vs lower tercile). Both unadjusted and age-adjusted models are presented.

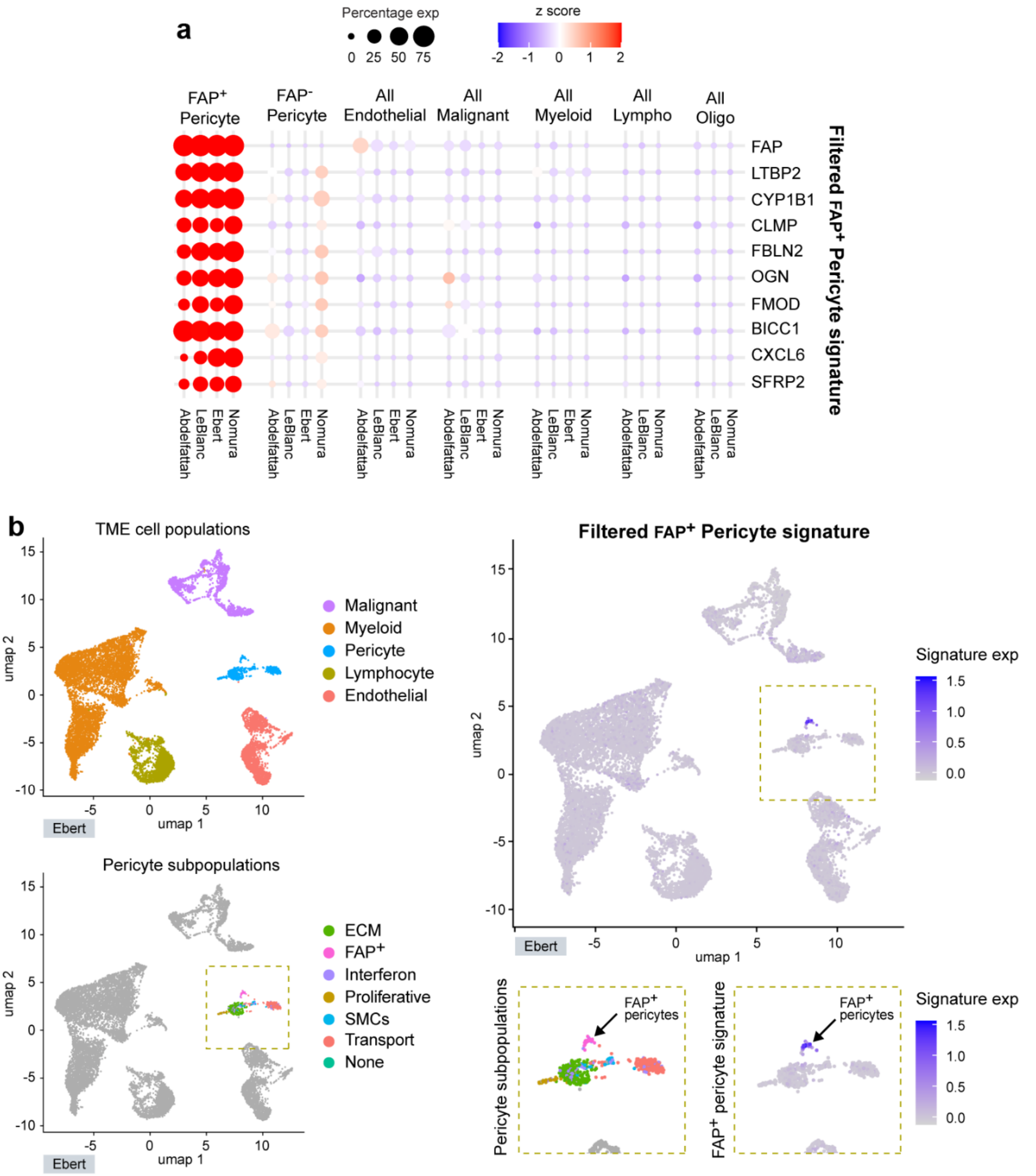

Supplementary Fig. 12. a Dotplots of FAP⁺ pericyte signature genes, filtered to remove genes highly expressed in other GBM tumor microenvironment cell types (see methods). Dot size indicates the percentage of expressing cells, and color intensity represents scaled expression (z score). Cell type annotations were adopted from the original publications. **b** Representative UMAPs of the Ebert dataset showing that high expression of the FAP⁺ pericyte signature is restricted to FAP⁺ pericytes.

References in supplementary figure legends:

1. Ebert, L. M. *et al.* Endothelial, pericyte and tumor cell expression in glioblastoma identifies fibroblast activation protein (FAP) as an excellent target for immunotherapy. *Clin. Transl. Immunology* **9**, e1191 (2020).
2. Abdelfattah, N. *et al.* Single-cell analysis of human glioma and immune cells identifies S100A4 as an immunotherapy target. *Nat. Commun.* **13**, 767 (2022).
3. LeBlanc, V. G. *et al.* Single-cell landscapes of primary glioblastomas and matched explants and cell lines show variable retention of inter- and intratumor heterogeneity. *Cancer Cell* **40**, 379-392. (2022).
4. Nomura, M. *et al.* The multilayered transcriptional architecture of glioblastoma ecosystems. *Nat. Genet.* **57**, 1155–1167 (2025).
5. Bejarano, L. *et al.* Single-cell atlas of endothelial and mural cells across primary and metastatic brain tumors. *Immunity* **58**, 1015-1032. (2025).
6. Ravi, V. M. *et al.* Spatially resolved multi-omics deciphers bidirectional tumor-host interdependence in glioblastoma. *Cancer Cell* **40**, 639-655. (2022).
7. Greenwald, A. C. *et al.* Integrative spatial analysis reveals a multi-layered organization of glioblastoma. *Cell* **187**, 2485-2501. (2024).
